# Cloaked Viruses and Viral Factors in Cutting Edge Exosome-Based Therapies

**DOI:** 10.3389/fcell.2020.00376

**Published:** 2020-05-26

**Authors:** Christos Dogrammatzis, Hope Waisner, Maria Kalamvoki

**Affiliations:** Department of Microbiology, Molecular Genetics, and Immunology, University of Kansas Medical Center, Kansas City, KS, United States

**Keywords:** exosomes, viral vectors, therapy, innate immunity, virus egress, exosomes biogenesis, miRNAs, cargo delivery

## Abstract

Extracellular vesicles (EVs) constitute a heterogeneous group of vesicles released by all types of cells that play a major role in intercellular communication. The field of EVs started gaining attention since it was realized that these vesicles are not waste bags, but they carry specific cargo and they communicate specific messages to recipient cells. EVs can deliver different types of RNAs, proteins, and lipids from donor to recipient cells and they can influence recipient cell functions, despite their limited capacity for cargo. EVs have been compared to viruses because of their size, cell entry pathways, and biogenesis and to viral vectors because they can be loaded with desired cargo, modified, and re-targeted. These properties along with the fact that EVs are stable in body fluids, they can be produced and purified in large quantities, they can cross the blood–brain barrier, and autologous EVs do not appear to cause major adverse effects, have rendered them attractive for therapeutic use. Here, we discuss the potential for therapeutic use of EVs derived from virus infected cells or EVs carrying viral factors. We have focused on six major concepts: (i) the role of EVs in virus-based oncolytic therapy or virus-based gene delivery approaches; (ii) the potential use of EVs for developing viral vaccines or optimizing already existing vaccines; (iii) the role of EVs in delivering RNAs and proteins in the context of viral infections and modulating the microenvironment of infection; (iv) how to take advantage of viral features to design effective means of EV targeting, uptake, and cargo packaging; (v) the potential of EVs in antiviral drug delivery; and (vi) identification of novel antiviral targets based on EV biogenesis factors hijacked by viruses for assembly and egress. It has been less than a decade since more attention was given to EV research and some interesting concepts have already been developed. In the coming years, additional information on EV biogenesis, how they are hijacked and utilized by pathogens, and their impact on the microenvironment of infection is expected to indicate avenues to optimize existing therapeutic tools and develop novel approaches.

## Introduction

Extracellular vesicles (EVs) are released by all cell types, including bacteria, archaea, fungi, and eukaryotes (Deatheragea and Cooksona, 2012). The properties and functions of these EVs have become a subject of recent investigation, as techniques for their isolation and characterization have advanced (Théry et al., 2006; Van Niel et al., 2018). EVs have diverse functions, including delivering selected cargo (such as MHC molecules) to stimulate T cells, transferring antigens to dendritic cells for cross-presentation to T cells, and delivering genetic materials such as transcripts and miRNAs that can cause epigenetic modifications to the cells (Théry et al., 2002).

Generally, EVs are classified based on their size and origin into three major groups, exosomes, microvesicles, and apoptotic bodies (Stoorvogel et al., 2002; Raposo and Stoorvogel, 2013; Kowal et al., 2014; Tricarico et al., 2017; Van Niel et al., 2018). All three classes of vesicles have different lipid composition, contain a wide variety of cellular components, such as DNA, coding and non-coding RNAs, proteins, and their size can vary from a few nm to μm. Thus EVs are considered to be highly heterogeneous (Van Niel et al., 2018).

There are multiple pathways involved in the biogenesis of EVs (Raposo and Stoorvogel, 2013; Tricarico et al., 2017; Van Niel et al., 2018). The Endosomal Sorting Complex Required for Transport (ESCRT) pathway has been implicated in the biogenesis of exosomes but ESCRT-independent mechanisms for exosomes formation also exist, as evidenced by the detection of vesicles containing CD63, a tetraspanin that mediates cargo sorting and intraluminal vesicles (ILVs) formation, but not ESCRT components (van Niel et al., 2011). The factors responsible for the production of plasma membrane-derived vesicles (microvesicles) are less well defined, but changes in membrane lipid composition at the budding sites and alterations in calcium levels have been reported (Piccin et al., 2007; Pap et al., 2009; Tricarico et al., 2017; Van Niel et al., 2018).

With the identification of biological roles for EVs, one emerging area is how the production and content of EVs may be modulated during infections and how these EVs could contribute to viral-mediated pathogenesis (Madison and Okeoma, 2015; Kalamvoki and Deschamps, 2016). It appears that there is an overlap between EV biogenesis and virion assembly or egress from the host cells (Gould et al., 2003; Wurdinger et al., 2012; Schorey et al., 2015; van Dongen et al., 2016; Sadeghipour and Mathias, 2017; Dogrammatzis et al., 2019). Moreover, changes in the cargo of EVs during infections could cause a variety of effects on uninfected recipient cells (Walker et al., 2009; Temme et al., 2010; Deschamps and Kalamvoki, 2018; Kakizaki et al., 2018). These observations have led to increased interest in characterizing EVs from infected cells and determining their possible implications in viral-mediated pathogenesis.

An additional aspect is how EVs could be used for therapeutic purposes, as they have some attractive qualities such as ease of isolation and the potential to be loaded with different molecules. Studies have also indicated the potential for EVs to serve as vaccines or adjuvants (Schorey et al., 2015). Additionally, it has been shown that during cancer progression there are changes to the numbers and cargo of EVs, which could have some diagnostic potential (Schorey et al., 2015; Jaiswal and Sedger, 2019). There have also been studies showing that antigen loading onto exosomes can stimulate an anti-tumor response (Zeelenberg et al., 2008; Tai et al., 2018). Also, delivery of EVs from pathogen infected cells can stimulate pro-inflammatory cytokine production in recipient cells (Bhatnagar and Schorey, 2007).

Despite the potential therapeutic applications for EVs, there is not yet an approved therapy. One issue is targeting of EVs to a particular tissue (Gyorgy et al., 2015). There are other concerns, such as the actual uptake of EVs by specific cell types, their activity, the ability to load EVs with a specific cargo, and the amount of EVs needed to achieve a desired effect while minimalizing off-target effects (Gyorgy et al., 2015).

Here, we discuss how viruses may help address some of the concerns regarding the use of EVs for therapeutic purposes. We discuss the potential use of EVs for drug delivery and their use for vaccine development against different viruses, as well as how some viruses or viral components could be delivered to treat different cancers. Moreover, we discuss how viruses could modulate the cargo of EVs, as well as the overlap in the biogenesis of EVs and virion morphogenesis. Finally, we summarize how understanding the interplay between viruses and EVs could be applied to the development of novel therapeutics and indicate novel potential targets.

## Ev Biogenesis Pathways Are Hijacked by Viruses for Their Assembly Indicating Novel Antiviral Targets

**FIGURE 1:**
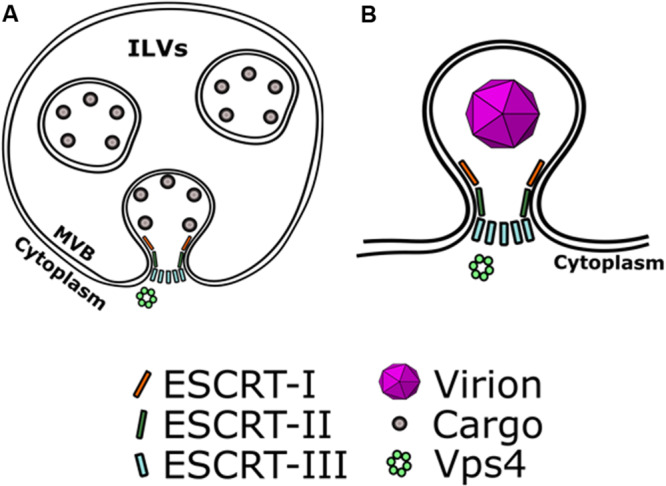
Viruses hijack EV biogenesis mechanisms to mediate their own assembly. The topology of viral envelopment requires constricting budding membranes toward the cytoplasm. **(A)** ESCRT recruits cargo on the membranes of early endosomes and can catalyze the formation of intra-luminal vesicles (ILVs) that contain ESCRT-recruited cargo. ESCRT-III catalyzes the scission of necks of ILVs. **(B)** In a similar manner, viruses can co-opt ESCRT to mediate their envelopment. ESCRT-III can catalyze scission of the budding neck of enveloping virions, allowing their release.

Extracellular vesicles constitute an effective means of cell-to-cell communication but often viruses co-opt EV biogenesis pathways for assembly and dissemination. For example, enveloped viruses can usurp EV biogenesis machinery to facilitate their assembly and envelopment at the plasma membrane or other membrane compartments (Lorizate and Kräusslich, 2011). The topology of virus budding from the cells requires constricting of the budding membranes toward the cytoplasm, and therefore factors that can catalyze membrane fission and can work from within the budding neck. The ESCRT pathway is known to perform a variety of membrane fission events, and such a capability may explain why it is utilized by different viruses (Figure 1). The best example to illustrate how the ESCRT pathway is utilized during virus budding is the process of HIV-1 assembly (Lorizate et al., 2013).

## Lessons From Viruses That Utilize ESCRT

**FIGURE 2:**
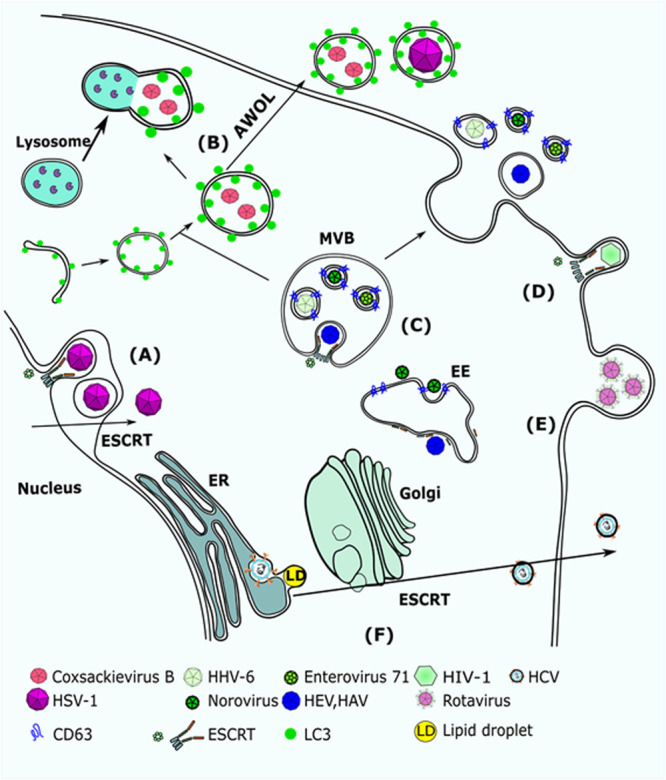
Viruses that utilize EV biogenesis mechanisms to mediate their release. **(A)** HSV-1 requires Alix and ESCRT-III for nuclear de-envelopment. Depletion of Alix results in accumulation of capsids in the internuclear space. **(B)** Coxsackievirus B (CVB3) was detected in autophagosome-like vesicles that were carrying LC3. It was suggested that CVB3 uses AWOL to increase its release from infected cells. HSV-1 was also suggested to use AWOL in oligodendroglial cells. **(C)** HHV-6, Norovirus, and EV71 were detected in CD63 + MVBs. **(D)** HIV-1 Gag recruits Alix to the plasma membrane, which mediates assembly of ESCRT-III and promotes scission of HIV virions. **(E)** Rotavirus was detected in large protrusions from the plasma membrane and in microvesicles that were larger than 500 nm. **(F)** HCV replicates in lipid enriched domains of the ER membrane and egresses through the Golgi to the plasma membranes. Extracellular release of HCV requires Hrs, a factor of the ESCRT-0 complex. AWOL, autophagosome-mediated exit without lysis; MVB, multi-vesicular body; EE, early endosome; ESCRT, endosomal complex required for transport.

Some viruses that hijack the ESCRT pathway encode proteins that contain one or more so-called “late domains.” These are peptide sequences that interact with proteins of the ESCRT pathway or ESCRT-associated components, and at least five distinct classes have been described. Such interactions have been best described for HIV-1 (Figure 2). HIV-1 Gag is the major viral structural protein. Gag is targeted to the inner leaflet of the plasma membrane by a bipartite targeting signal, and can capture the viral RNA genome and assemble into a spherical virion (Bharat et al., 2012). Late domains on HIV-1 capsid protein Gag [PPXY, P(S/T)AP and LYPXL] bind to ESCRT-I and Alix and mimic the ESCRT-0-ESCRT-I interaction. In parallel, Alix also binds CHMP4 and activates ESCRT-III assembly. ESCRT-III filaments were found to surround Gag assemblies at the plasma membrane in Vps4 depleted cells. Recent super-resolution studies show ESCRT proteins at the base of or inside budding virion necks, which suggests scission by ESCRT-III and Vps4. Further support for the role of ESCRT-III and Vps4 in scission of HIV-1 is provided by live imaging, which showed that exogenous GFP-tagged ESCRT-III and Vps4 were transiently recruited to budding HIV-1 particles just prior to their release. Looking also into other viruses, it becomes clear that viruses can enter the ESCRT pathway using late domains that can bind to different ESCRT accessory factors (e.g., Tsg101, Alix, and Nedd4) (Votteler and Sundquist, 2013).

Interestingly, non-enveloped viruses can also utilize ESCRT for their release, blurring the line between non-enveloped and enveloped viruses. Feng et al. (2013) demonstrated that hepatitis A virus (HAV) exits the cell in two forms; as a non-enveloped capsid that is readily detectable in a capsid antigen enzyme-linked immunosorbent assay (ELISA) and as a virion that is cloaked in host-derived membranes, thereby protecting the virion from antibody-mediated neutralization. They show that enveloped HAV (eHAV) constitutes 79% of virus released from infected cells. Release of eHAV seems to be dependent on ESCRT-associated proteins, but not on the entire ESCRT machinery (Figure 2). Anti-Alix antibodies precipitated encapsidated viral RNA, while silencing of Alix expression blocked release of the enveloped virus. Additionally, depletion of VPS4B leads to a significant reduction in eHAV release. A search for potential late domains in the HAV polyprotein identified two tandem YPXL motifs in the VP2 capsid protein, which mediates interactions with Alix in other cases of enveloped viruses. Tyr-to-Ala substitutions within either motif abrogated capsid assembly. These data are consistent with a role for the ESCRT machinery, the ESCRT accessory protein Alix, and the VP2 late domains in viral assembly and release. These results also revealed a previously unrecognized strategy by which a virus cloaks itself in host membranes to evade neutralizing antibodies. However, the picture was complicated by further work (González-López et al., 2018). It was shown that the YPXL motifs of HAV VP2 are redundant for Alix recruitment, and they identified mutations that inhibit virus release but not virus assembly. Also, the fact that these VP2 mutants are solvent-inaccessible considering the crystal structure of VP2, should restrict access of ESCRT factors, raising questions about the structure of the HAV capsid prior to and following envelopment.

Nagashima et al. (2017) worked with the Hepatitis E Virus (HEV), another non-enveloped virus, and in parallel with Emerson et al. (2010) they demonstrated that release of HEV from cultured cells depends on the ORF3 protein and requires an intact PXXP motif in the ORF3 protein. This motif mediates the interaction with TSG101 and facilitates the budding of membrane-associated HEV particles.

Tamai et al. (2012) showed that HCV secretion from host cells requires the Hrs-dependent exosomal pathway Figure 2. Hrs (Hepatocyte Growth Factor-regulated Tyrosine Kinase Substrate) is a component of the ESCRT pathway that recognizes ubiquitinated cargo and directs it to ILVs. Hrs depletion attenuated both HCV release into the supernatant and the number of infectious particles in the cytoplasm, but not the amount of HCV-RNA in the cytoplasm, suggesting that Hrs is involved in HCV assembly. The core protein (capsid) and the E2 (envelope) were found in MVBs in Huh7 cells, indicating that HCV probably takes advantage of the MVBs for its assembly. It is not clear how HCV utilizes ESCRT for its assembly though, as no canonical late domains exist in HCV proteins. Nonetheless, the HCV core has been reported to be ubiquitinated by E6AP/UBE3A (an E3 ubiquitin ligase). It is possible that Hrs can recognize the ubiquitinated HCV core protein and sort it to the viral budding site in the MVBs. Further work on HCV in MVBs has been done (Elgner et al., 2016) where a MVB formation inhibitor (U18666A) was used to investigate the relevance of MVBs/late endosomes to HCV virion morphogenesis and release. U18666A works by inhibiting the function of the NPC1 protein, a protein first described in Niemann–Pick disease patients. NPC1 inhibition abrogates the trafficking of cholesterol and results in its accumulation in large lysosomal structures, which can prove fatal. Under treatment with U18666A, significant inhibition of HCV release was observed, but the assembly of the particles was not affected, as shown by the retention of infectious particles inside the cells. Furthermore, in U18666A-treated cells HCV core accumulates in exosomal and autophagosomal structures, reflecting the involvement of the exosomal pathway in the release of HCV particles. Interestingly, HCV particles that are released in exosomes can be infectious *in vitro* (Ramakrishnaiah et al., 2013).

Late domains are not the only sorting signal that viruses can utilize to hijack ESCRT. Proteins that are ubiquitinated can be recognized by the Hrs (ESCRT-0) component, the first step in the ESCRT pathway. Binding of Hrs to ubiquitinated cargo can recruit the ESCRT-I complex, which then recruits the ESCRT-II and -III complexes. Ubiquitin depletion has been shown to inhibit virus budding (Votteler and Sundquist, 2013), and ubiquitin itself can recruit ESCRT components when conjugated to retroviral Gag proteins (Joshi et al., 2008). Additionally, multiple components of ESCRT contain ubiquitin binding domains (Bissig and Gruenberg, 2014; Olmos and Carlton, 2016) and decreased viral budding can be observed when forms of ubiquitin, which lack the ability to form K63-linked chains, are overexpressed (Strack et al., 2002).

## Strategies Developed by Viruses That Do Not Utilize ESCRT Pathways

Viruses can also utilize ESCRT-independent EV biogenesis pathways as a means of dissemination or assembly and envelopment (Figure 2). Most often, ESCRT independence is inferred from insensitivity to knockdown of the Vps4 ATPase (the recycling factor of ESCRT). It is unclear what cues the viruses use to hijack the host EV biogenesis machinery, and most work focuses on demonstrating the shedding of virions inside vesicles of plasma membrane (PM) or endosomal origin.

Enteroviruses seems to utilize both vesicles of PM and endosomal origin to assemble and disseminate. Santiana et al. (2018) show that rotaviruses and noroviruses are shed in non-negligible quantities inside EVs and have a disproportionately larger contribution to infectivity than free viruses. They detected rotaviruses inside protrusions from the plasma membrane that is consistent with rotavirus release in microvesicles (Figure 2). Interestingly, rotaviruses in microvesicles were also detected in stool samples. Microscopic analysis of vesicles isolated from stool samples confirmed the presence of viruses inside large EVs, with 70% of them being >500 nm. On the other hand, noroviruses were detected in vesicles of exosomal origin, as shown by EM of the norovirus-containing vesicles, and further confirmed by the presence of the tetraspanins CD63, CD81, and CD9, and by inhibition of exosome biogenesis through GW4869 treatment, a neutral sphingomyelinase inhibitor that inhibits production of ceramide, which is a major structural component of exosomes. Although both rotaviruses and noroviruses seem to exploit the EV biogenesis pathways for their own dissemination, it remains undetermined what viral cues are utilized to target the virions in exosomes or microvesicles.

Coxsackievirus B3 (CVB3) is another enterovirus shedding inside microvesicles. Robinson et al. (2014) studied the dissemination of Coxsackievirus and visualized the route of infection. They utilized a recombinant CVB3 expressing “fluorescent timer” protein (Timer-CVB3), which “develops” from green to red and is used to distinguish recently infected from previously infected cells. Infection of partly differentiated neural progenitor and stem cells (NPSCs) and C2C12 myoblast cells induced the release of abundant extracellular microvesicles (EMVs) containing red Timer-CVB3 and infectious virus. Virions were also observed in EMVs by transmission electron microcopy. Interestingly, the lipidated form of LC3 was detected in released EMVs that harbored infectious virus, suggesting that the autophagy pathway may play a role in EMV shedding (Figure 2). This pathway may be similar to the means of extracellular delivery of poliovirus (Taylor et al., 2009). Infection with poliovirus induced autophagosome-like vesicles that harbor poliovirus particles. Taylor et al. (2009) proposed that this extracellular delivery of cytoplasmic contents be termed autophagosome-mediated exit without lysis (AWOL), and this might be utilized by CVB3 as shown by Robinson et al. (2014).

Mao et al. (2016) suggest that Enterovirus 71 (EV71) can shed inside exosomes from EV71-infected cells, and those virus-containing EVs can establish a productive infection in human neuroblastoma cell lines (SK-N-SH). EV71 RNA and the VP1 capsid protein have previously been detected in extracellular vesicles, which supports EV71 utilization of EVs. Mao et al., defined the EVs they isolated as exosomes based on CD63 (Figure 2).

Herpesviruses also utilize ESCRT-independent pathways to mediate their assembly and egress. Mori et al. (2008) suggested that human herpes virus-6 (HHV-6) is released in vesicles produced from the exosomal pathway (Figure 2). First, they analyzed the intracellular localization of the HHV-6 structural components, such as the envelope protein gB, by immunofluorescence microscopy during the late stages of infection. They found that gB colocalized partially with CD63 in the juxtanuclear area, indicating that it was associated with late endosomes. Looking into the maturation pathway of HHV-6 by EM, they detected vesicular or tubular structures that surrounded virions, and were distinct from the Golgi network. Those structures were also partially positive for CD63, indicating that the virus enveloping membrane may have characteristics shared with TGN and endosomes. Furthermore, structures resembling MVBs were found containing virions and small vesicles, which were fused with the PM and released both virions and vesicles in the extracellular space. These results suggest that HHV-6 hijacks the EV biogenesis machinery to mediate its envelopment and release.

Bello-Morales et al. (2018) worked on HSV-1 in oligodendrocytes (OLs). OLs are the myelin-forming cells of the central nervous system (CNS) and are highly susceptible to HSV-1 infection. Bello-Morales et al. (2018) suggested that HSV-1 can be packaged in EVs from OLs and transferred from infected to uninfected cells. Using electron microscopy approaches (TEM), they detected microvesicles (MVs) carrying HSV-1 virions (Figure 2). In their case the term microvesicles was used to describe vesicles of unknown origin, rather than vesicles released from the plasma membrane. They also found that Chinese hamster ovary (CHO) cells, which lack receptors for HSV-1, were susceptible to HSV-1 infection after exposure to virus-containing MVs that were isolated from the supernatant of infected OL cells. Incubation of virus-containing MVs with anti-HSV-1 antibodies did not neutralize infection of the CHO cells. Therefore, they proposed that packaging of HSV-1 in MVs from OLs may be a means of virus spread by avoiding immune surveillance. The exact mechanistic process of targeting HSV-1 to MVs remains unclear though. The fact that MVs released by OLs are LC3-II positive suggests that autophagic processes contribute to viral shedding in a manner similar to AWOL, proposed by Taylor et al. (2009) and Robinson et al. (2014) However, this would require further work to confirm that the MVs that carry virus are LC3-II positive, since Bello-Morales et al. (2018) did not perform any single-EV analysis. Additionally, other work (Pawliczek and Crump, 2009) suggests that HSV-1 utilizes the ESCRT-III pathway to mediate its envelopment, but is independent of Alix and Tsg101, whereas Arii et al. (2018) suggest that HSV-1 utilized ESCRT and Alix for its nuclear envelopment and de-envelopment. The mechanistic details of HSV-1 utilizing ESCRT or MVs are not clear, as late domains interacting with ESCRT have not been described for HSV-1 proteins. However, ESCRT might facilitate HSV-1 assembly through recognizing ubiquitinated capsid proteins. HSV-1 encodes an E3 ubiquitin ligase (ICP0) and a de-ubiqutinase (UL36) to modify host ubiquitin functions and could hijack the ESCRT pathway by regulating the ubiquitination of host and viral factors. For example, it was demonstrated that in the case of HSV-2, the viral tegument protein UL56 regulates Nedd4 ligase localization in the cytoplasm and thereby could mediate viral egress (Ushijima et al., 2008).

## The Viral Modified Proteinaceous Cargo of EVs Dictates Novel Therapeutic Targets

With the discovery that some viruses hijack EV biogenesis pathways to be released from infected cells, more work has focused on if exosomes released from infected cells can carry viral proteins or if viral infections can modify the proteinaceous cargo of EVs. An example of a virus infection where released EVs contain viral proteins is the observation that fibroblasts infected with human cytomegalovirus release EVs that contain certain viral glycoproteins and this was independent of free virus (Zicari et al., 2018). Similar data were obtained several years ago by Temme et al. (2010) who demonstrated that HSV-1 modifies the cargo of EVs through the functions of the glycoprotein B (gB). This group found that in cells transfected with gB, the EVs released contained gB and the human leukocyte antigen, isotype DR (HLA-DR), however, HLA-DR is typically sorted into compartments with MHC class II where it encounters processed antigens that are then presented on the cell surface (Temme et al., 2010). These data suggest that HSV-1 is able to prevent this antigen presentation through instead sorting HLA-DR into EVs with gB (Temme et al., 2010).

**FIGURE 3:**
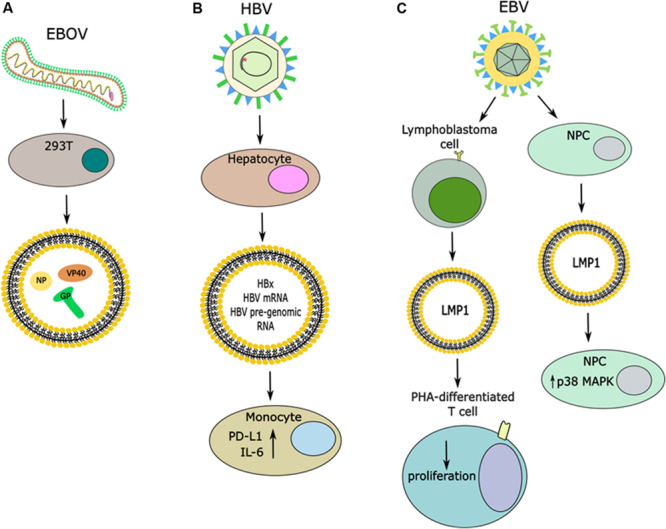
Release of viral proteins in EVs and effects in recipient cells. **(A)** Infection with EBOV causes the release of vesicles that contain viral proteins, including the NP, GP, and VP40. **(B)** Infection of hepatocytes with HBV induces the release of vesicles that contain the HBx protein, it’s mRNA and HBV pre-genomic RNA. Uptake of these vesicles by monocytes can cause increases in expression of PD-L1 and IL-6. **(C)** Infection of multiple different cell types with EBV has been shown to induce the release of LMP1 in EVs. When these EVs are taken up by PHA-differentiated T cells it caused decreased proliferation, while uptake of LMP1 + EVs by NPCs caused an increase in p38 MAP kinase signaling.

In another study, it was discovered that exosomes released from hepatocytes transfected with full length HCV genome containing Flag-tagged E2 could release exosomes containing E2, which was independent of viral genome replication or the core protein (Deng et al., 2019). Ebola virus (EBOV) is another example of a virus where several viral proteins have been detected in exosomes from infected or transfected cells, including VP40, NP, and GP proteins (Figure 3A) (Pleet et al., 2016, 2019). The most well-studied so far is VP40, the matrix protein, and it was found that exosomal VP affects the RNAi machinery in recipient cells and causes cell death in immune cells, such as T cells, B cells, and monocytes (Pleet et al., 2016, 2018). Moreover, VP40 has been found to modulate the cell cycle, in part through binding to the promoter of cyclin D1 and increasing its levels (Pleet et al., 2018). It seems that this function of VP40 also regulates when EVs are released, with the most EVs released in G1/S or G2/M phases (Pleet et al., 2018). This group also found that there was differential expression of various cytokines in EVs from VP40-expressing cells, many of which have previously been identified to exacerbate EBOV pathogenesis (Pleet et al., 2018, 2019). While the nucleoprotein, NP, of EBOV is known to bind to viral RNA, it has been shown to not be specific for only viral RNA and therefore its role in EVs should be further explored (Noda et al., 2010; Pleet et al., 2019). It is not yet known what potential roles there are for the GP protein of EBOV, but it is known to be required for viral entry into the host cell and is trafficked through the ER and Golgi vesicular transport system (Pleet et al., 2019). Therefore, more work should be done to characterize the roles for both NP and GP in exosomes from EBOV-infected cells. Additionally, the presence of these proteins in exosomes may represent a novel therapeutic target. These results have not been explored for their effect on recipient cells, nor their contribution to pathogenesis or therapeutic potential. Overall, these are just a few pieces of evidence that viral infections alter the protein cargo released in EVs. Additionally, viral infection can trigger changes to host proteins released in exosomes. For instance, HSV-1 infections triggers the release of the innate immunity DNA sensor, STING, in exosomes which can stimulate immune responses in uninfected recipient cells and down-regulate a subsequent HSV-1 infection (Kalamvoki et al., 2014; Deschamps and Kalamvoki, 2018). Therefore, it seems that viral infections can have varying effects on the protein cargo of EVs.

Using tick-borne Langat virus (LGTV), a model flavivirus similar to tick-borne encephalitis virus (TBEV), one group has found that this virus induces the release of exosomes from tick cells and human brain endothelial cells that contain viral proteins and intact viral genome, which can then be transported and taken up by neuronal cells (Zhou et al., 2018). I nfected neuronal cells were also found to release exosomes containing LGTV RNA and proteins (Zhou et al., 2018). These researchers found similar results when culturing mouse cortical neurons and infecting with LGTV, where blocking exosome release led to decreased infection of cortical neurons (Zhou et al., 2018). These findings could be used to develop a novel therapeutic strategy for targeting TBEV. Moreover, these findings should be further explored to understand how EVs cross the blood–brain barrier and the impact they could have on other cell types in the brain that may take up these EVs.

One study has found that the HBV mRNA for the protein HBx can be exported in exosomes from cells expressing the HBx protein, and through a mass spectrometry screening it was found that the HBx protein is present in exosomes as well (Figure 3B) (Kapoor et al., 2017). Furthermore, this group saw that when exposing exosomes containing HBx to hepatic stellate cells it led to expression of various transactivators and proliferation proteins, which was not seen from control exosomes (Kapoor et al., 2017). This study warrants further investigation of the role of HBx protein in exosomes and its potential role in disease progression and viral-mediated pathogenesis. From an immunology perspective, another group found that EVs released from cells containing pre-genomic RNA of HBV under an inducible promoter (HepAD38 cells) had an immunosuppressive function when exposed to monocytes from patients, as evidenced by increased PD-L1 expression (Figure 3B) (Kakizaki et al., 2018). This group also found that this effect was partially reversed when HepAD38 cells were stimulated to express HBV and exposed to nucleotide reverse transcriptase inhibitors (NRTIs) prior to isolating EVs (Kakizaki et al., 2018). Another group has found that in an HBV-inducible cell line the protein cargo of EVs from uninduced cells versus induced cells differed (Jia et al., 2017). One major difference noted was down-regulation of multiple subunits of the 26S proteasome complex from induced cells when compared to uninduced cells (Jia et al., 2017). Furthermore, when EVs from uninduced cells or induced cells were incubated with human peripheral monocytes, higher IL-6 (a pro-inflammatory cytokine) production was observed from cells incubated with EVs from the HBV-induced cell line (Figure 3B) (Jia et al., 2017). Therefore, the immunological impact of EVs released from HBV infected cell lines warrants further investigation as it implies a role in disease progression. These examples further bolster that viral-mediated pathogenesis is a complex process that involves both intra- and inter-cellular signaling.

Another viral protein that is secreted in exosomes is the HIV Nef (Raymond et al., 2011; Lee et al., 2016). One group examined the role of Nef-containing EVs on macrophage recipient cells (Mukhamedova et al., 2019). These researchers produced Nef-containing EVs and found that they were able to reorganize lipid rafts in recipient macrophages, including altering the lipid composition, increasing lipid rafts abundance, and reducing the abundance of ABCA1 (the lipid transporter, ATP binding cassette transporter type A1) in lipid rafts (Mukhamedova et al., 2019). They found that this reorganization of lipid rafts functions to re-locate the inflammatory receptor TLR4 and the inflammatory response amplifier TREM-1 to lipid rafts, which indeed led to increased inflammation in macrophages pre-treated with exosomal Nef and then treated with LPS (Klesney-Tait et al., 2006; Mukhamedova et al., 2019). Additionally, these researchers administered exosomes containing Nef to mice intravenously and saw recapitulation of their *in vitro* results, as evidenced by increased lipid rafts in monocytes and increased plasma levels of pro-inflammatory cytokines (Mukhamedova et al., 2019). Finally, these results were also observed through use of HIV infected human macrophages or plasma from HIV-positive patients, where exposure of macrophages to Nef-containing vesicles and treatment with LPS recapitulated the *in vitro* results and mouse experiment results (Mukhamedova et al., 2019). These findings demonstrate a novel understanding of the role of exosomal Nef and its potential role in disease progression.

In another example, it was found that in both asthmatic and non-asthmatic primary bronchial epithelial cells (PBECs), infection with either of two serotypes of human rhinovirus (RV) induced the extracellular release of tenascin-c (TN-C) in a cell death-independent manner (Mills et al., 2019). TN-C is an extracellular matrix glycoprotein and its expression is positively correlated with asthma in humans as it sustains chronic inflammation (Mills et al., 2019). This group demonstrated that when PBECs were treated with poly(I:C), a sequence of nucleic acids that mimics the virus, TN-C was released in EVs and when these EVs were exposed to epithelial cells or macrophages there was increased expression of pro-inflammatory cytokines which may explain how RV exacerbates asthma (Mills et al., 2019). These findings could be expanded upon to further examine cargo changes during RV infection, as well as understand how other co-morbidities may affect the cargo of EVs.

In an additional example, cells infected with human papillomavirus (HPV), specifically HeLa cells with detectable expression of the HPV oncogenes E6/E7, release the anti-apoptotic factor Survivin into the extracellular environment in EVs, which leads to increased cell proliferation, decreased pro-apoptotic factor expression, and may aid in cancer cell migration following uptake (Khan et al., 2009; Khan et al., 2011; Honegger et al., 2013). This group also found that a mutant of Survivin, the Surv-T34A that can no longer block apoptosis, also was released into the extracellular environment and caused apoptosis in recipient cells (Khan et al., 2009). This pro-apoptotic effect was exacerbated when chemotherapy drugs were combined with exposure of cells to Surv-T34A (Khan et al., 2009). This observation represents a potentially novel therapeutic strategy to induce apoptosis in cancer cells.

Furthermore, it was found that in Epstein-Barr virus (EBV)-positive lymphoblastoma cell lines, which express the viral oncogene latent membrane protein (LMP1), LMP1 was released in exosomes (Figure 3C) (Flanagan et al., 2003). This group differentiated peripheral blood mononuclear cells (PBMCs) with the mitogen phytohemagglutinin (PHA) to induce a T-cell phenotype and then exposed the cells to LMP1-positive exosomes, where they observed decreased T cell proliferation (Figure 3C) (Flanagan et al., 2003). Another group utilized these findings to expose nasopharyngeal carcinoma cells (NPCs) to exosomes from NPCs containing LMP1, where they observed induction of p38 MAP kinase signaling (Figure 3C) (Zhang et al., 2019). This signaling led to increased cell proliferation and increased resistance of NPCs to ionizing radiation, as evidenced by decreased apoptosis upon exposure to radiation (Zhang et al., 2019). Altogether, these data suggest a role of exosomal LMP1 in viral-mediated pathogenesis.

The previous examples indicated novel research directions relevant to disease pathogenesis. One group has attempted a novel therapeutic approach based on delivering selected factors in EVs with some promising results. This group engineered EVs to deliver anti-HIV Env antibodies with either an apoptosis-inducing miRNA (miR-143) or the chemotherapy compound curcumin, which has been shown to have anti-HIV activity, as well as anti-inflammatory and anti-tumor effects (Liang et al., 2016; Mantzorou et al., 2018; Tan et al., 2019). This group found that they could indeed target Env-expressing cells with exosomes containing the anti-Env antibody and if the exosomes also contained curcumin, the Env-positive cells died (Zou et al., 2019). Worth noting is that exosomes containing anti-Env and curcumin also caused some cell death in Env-negative cells, though it was less than what was seen in Env-positive cells. Similarly, when exosomes were loaded with anti-Env and miR-143 they observed about 60% cell death in Env-positive cells, while also observing about 35% cell death in Env-negative cells (Zou et al., 2019). Furthermore, treating HIV-infected cells with exosomes containing anti-Env and either curcumin or miR-143 suppressed the viral infection while also killing the infected cells (Zou et al., 2019). This effect with exosomes containing anti-Env and either curcumin or miR-143 was also observed in cell lines where the virus was latent and then re-activated, or in PBMCs collected from chronically infected patients undergoing antiretroviral therapy (ART) (Zou et al., 2019). Finally, these researchers engineered an Env-expressing tumor model to represent the bodily reservoir of HIV during latency. When exosomes containing anti-Env and curcumin were injected intravenously in mice, they saw inhibition of tumor growth indicating a potential use for engineered EVs in treating latent HIV (Zou et al., 2019). Optimizing this approach for more efficient targeting of Env-positive cells will open novel avenues to treat virally infected cells, and this strategy could subsequently be modified to target cancer cells.

The cumulative evidence presented here represents a multitude of proteins, viral and host, that could serve as therapeutic targets or could be utilized for therapeutic purposes, but this will require further investigation.

## The Therapeutic Potential of Host miRnas Released From Virus Infected Cells

In 2007, it was first described that multiple RNA species can be carried (or shuttled) into EVs that could be delivered to recipient cells, and microRNAs (miRNAs) were among the species present in EVs (Valadi et al., 2007). There have been numerous studies regarding the role of extracellular miRNAs in cell-to-cell communication, as well as the presence of extracellular miRNAs in various cancers (Mitchell et al., 2008; Skog et al., 2008; Taylor and Gercel-Taylor, 2008; Barger et al., 2016). It has even been established that delivery of miRNAs in exosomes could be used as a therapy for some cancers. In one such study, miR-124a was delivered with a lentiviral vector to mesenchymal stem cells, where miR-124a was then packaged into EVs (Lang et al., 2018). Upon delivery of EVs containing miR-124a to glioma stem cell lines, the survival of cancer cells was reduced and when glioma stem cells were pre-treated with exosomal miR-124a and then implanted into mice, it was found that mice survived longer compared to mice implanted with untreated glioma stem cells (Lang et al., 2018). In one form of prostate cancer it was found that miR-146a was down-regulated and when it was exogenously expressed in a prostate cancer cell line it reduced the expression of its target gene, ROCK1, by almost 80% and consequently reduced cell proliferation, invasion, and metastasis (Lin et al., 2008). This work was then expanded upon using extracellular miR-146a produced from fibroblasts. The supernatant from these cells was exposed to a prostate cancer cell line, where down-regulation of ROCK1 was again observed and cell proliferation was reduced (Kosaka et al., 2010). It should be noted that this group did not confirm that miR-146a was in exosomes specifically, but this work is an example of how miRNAs can be secreted from cells and used as a potential therapy for treatment of certain types of cancers.

With these promising initial results, research has been done looking at how viruses modify the miRNA cargo of EVs and identifying novel therapeutic targets. EBV down-regulates some cellular miRNAs. Particularly, it was found that in EBV-associated gastric carcinoma the cellular miRNAs, miR-200a and miR-200b, were downregulated in patient tissue samples (Shinozaki et al., 2010). Down-regulation of miR-200a and miR-200b leads to the down-regulation of E-cadherin expression, which is a critical step in the development of EBV-associated gastric carcinomas (Shinozaki et al., 2010). Therefore, restoring expression of miR-200a and miR-200b would be inhibitory on gastric carcinogenesis, and indeed with overexpression of other members of this miRNAs family one group observed inhibition of cancer cell proliferation (Du et al., 2009). These findings could be used to develop a therapeutic approach based on restoring the levels of miR-200a or miR-200b through EV delivery.

Not only is EBV known to modify the miRNA cargo of EVs, but other viruses are known to affect the RNA cargo of EVs as well. Recently, it was found that during respiratory syncytial virus (RSV) infection of lung carcinoma cells there are changes to the composition of RNA species in exosomes, including miRNAs and piwi-interacting RNAs (piRNAs), compared to uninfected cells (Chahar et al., 2018). When exosomes were isolated from RSV infected cells and then exposed to either human monocytes or uninfected lung carcinoma cells pro-inflammatory cytokines were upregulated (Chahar et al., 2018). These data suggest that exosomes from RSV infected cells contain cargo that can induce an immune response, and this is perhaps due to the altered RNA species that are carried by these exosomes. These exosomes from RSV infected cells could then have therapeutic potential.

**FIGURE 4:**
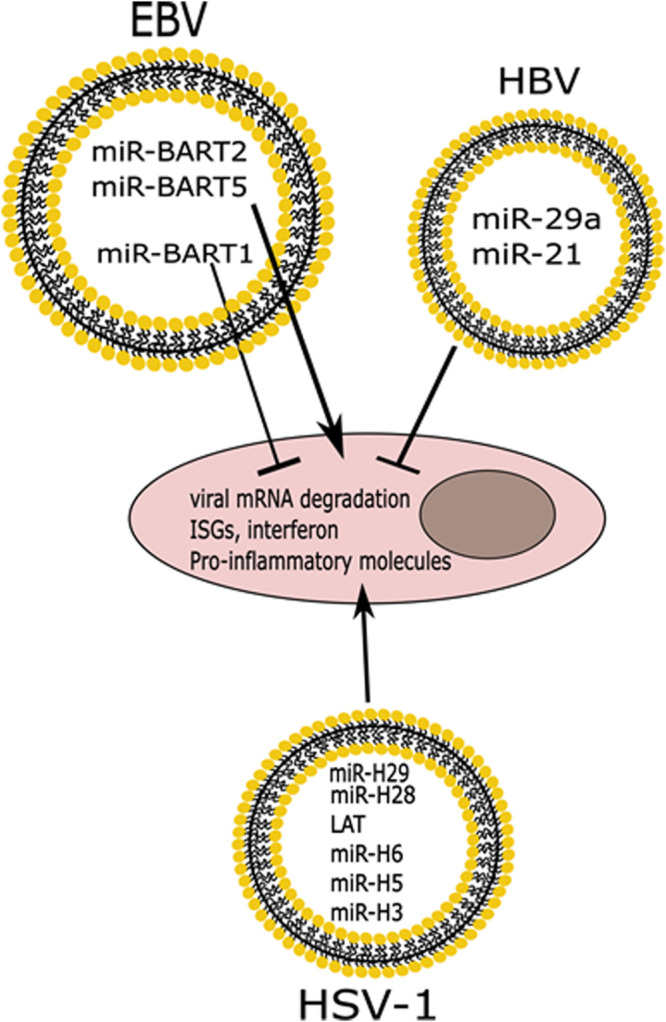
Differential effects of miRNAs released in EVs from infected cells. Infection with HSV-1 or EBV induces the release of vesicles containing viral miRNAs that cause degradation of viral mRNAs, while also inducing the expression of ISGs and pro-inflammatory molecules. EBV also expresses a miRNA that functions to block innate immune responses. HBV infection induces the release of immunomodulatory miRNAs that also suppress the innate immune response.

Another group found that hepatitis B virus (HBV) infection of hepatocytes increased the abundance of the immunomodulatory miRNAs, miR-21 and miR-29a, in EVs, which led to a decrease in the mRNA targets of these miRNAs in THP-1 macrophages exposed to these EVs (IL-12p35 and IL-12p40, respectively) (Figure 4) (Kouwaki et al., 2016). This evidence can be used to better understand how HBV suppresses the immune response during chronic infection and could be exploited for therapeutic uses (Kouwaki et al., 2016). It was found by another group that patients with chronic HBV infection (CHB) [whose levels of alanine aminotransferase (ALT) were normal, while they did have liver tissue inflammation] had changes in the secretion of miRNAs in exosomes depending on the severity of liver tissue inflammation, as evidenced by high throughput sequencing (Ronghua et al., 2018). This represents a novel method to monitor CHB that does not rely on detection of ALT, which is classically used as a method to detect liver disease, and could also be used to determine severity of liver inflammation (Ronghua et al., 2018).

Hepatitis C virus (HCV) has also been shown to alter the miRNA cargo of EVs. It was previously demonstrated that there is an association of the viral RNA with Ago2, miR-122, and heat shock protein 90 (HSP90) and that this has a positive effect on viral replication (Henke et al., 2008; Wilson et al., 2011; Bukong et al., 2013). MicroRNA-122 is expressed primarily in liver cells and is important for stability, translation, and replication of HCV genome (Henke et al., 2008). These findings were then used to examine the cargo of exosomes from either patient samples of primary hepatocytes or from a liver cell line, where it was found that in both cases exosomes contain a complex of HCV RNA with Ago2, HSP90, and miR-122 (Bukong et al., 2013). It was also demonstrated that after EV uptake a productive HCV infection occurs independent of free virus (Bukong et al., 2014). Therefore, miR-122 may represent a novel therapeutic target for blocking HCV infection.

Regarding HPV, it was found that there is differential expression of about 10 intracellular miRNAs in E6/E7 positive compared to E6/E7 negative cells (Honegger et al., 2015). This group also found that there were seven distinct miRNAs abundant in exosomes released by HeLa cells that appear to have roles in cellular proliferation and apoptosis, and thus positively impact HPV-positive cancer cell growth (Honegger et al., 2015). Aside from the aberrant expression profiles of miRNAs in HPV-positive cells and their exosomes, there is still much to be explored about these miRNAs and their potential as therapeutic targets.

## Therapeutic Potential of Herpesvirus miRnas Released Into Exosomes

Members of the *Herpesviridae* family are well known for expressing miRNAs (Skalsky and Cullen, 2010). It has been shown that several EBV miRNAs, specifically miR-BART2-5p, miR-BART7-3p, miR-BART13-3p, and miR-BART1-5p, are found in the circulation of patients who have EBV-associated nasal natural killer/T-cell lymphoma (NNKTL), which is a disease with poor prognosis (Komabayashi et al., 2017). The levels of these miRNAs decreased after patients underwent treatment, which suggests that detection of these EBV miRNAs could be used as biomarkers for NNKTL (Komabayashi et al., 2017).

It was also found that the BART1 miRNA of EBV is exported in EVs, and it is known to target immunomodulatory peptides such as CXCL11 in uninfected cells (Figure 4) (Pegtel et al., 2010). This group also showed that other EBV miRNAs such as miR-BART2 and miR-BART5 which target the EBV DNA polymerase transcript and the viral LMP1 transcript, respectively, are found in exosomes (Figure 4) (Pegtel et al., 2010). HSV-1 miRNAs are expressed mainly during latency and some of the targets include the transcripts for the immediate early genes, ICP0 and ICP4, and the transcript for ICP34.5, all of which are important for a successful productive infection (Skalsky and Cullen, 2010). There has been a lot of interest in understanding how HSV-1 miRNAs can affect the infection. Interestingly, some of the HSV-1 miRNAs have been found in exosomes released by infected cells (Kalamvoki et al., 2014; Han et al., 2016; Wang et al., 2018; Huang et al., 2019). In one such study, researchers developed artificial miRNAs targeting the transcript of the ICP4 gene of HSV-1, an immediate-early gene that encodes for the essential regulatory protein ICP4 (Wang et al., 2018). These researchers found that an exosomal miRNA targeting ICP4 could block a subsequent HSV-1 infection, and were able to optimize packaging of this miRNA by adding an exosome targeting sequence to it (Wang et al., 2018). Additionally, it was found that two HSV-1 miRNAs, miR-H28 and miR-H29, are released into EVs during the late stage of the productive infection and if they are exposed to cells during early times post-infection they can lead to a reduction in viral yields (Figure 4) (Han et al., 2016). Building on this, the same group found that exposing cells to miR-H28 caused an increase in IFN-γ production, which restricts the virus (Huang et al., 2019). Kalamvoki et al. (2014) have also found that LAT, miR-H3, miR-H5, and miR-H6 are all exported in EVs from HSV-1 infected cells, and the role for these miRNAs in recipient cells remains to be characterized (Figure 4). The seminal implication of these findings is that a potential therapeutic approach could be developed in which EVs are engineered to contain viral miRNAs that could control HSV-1 infection and dissemination.

Cumulative data described here suggest multiple roles for both host and viral miRNAs in EVs that may be beneficial from a therapeutic perspective. Overall, the role viruses play in modifying the secretion of host miRNAs is of interest to better understand interactions between the virus and the host.

## Therapeutic Potential of Antiviral Factors Delivered by Extracellular Vesicles

Autologous EVs have negligible immunogenicity when administered *in vivo* (Alvarez-Erviti et al., 2011), and can enhance the stability and biodistribution of their cargo (Sun et al., 2010; Shtam et al., 2018). EVs can be loaded with desired molecules either *ex vivo* or during their biogenesis. EVs in the literature have been loaded with miRNAs, shRNAs, mRNAs, proteins, and small molecules (Zhang et al., 2016; Hurwitz et al., 2017; Anticoli et al., 2018; McNamara et al., 2018). There are many publications regarding the use of EVs as a vehicle for drug delivery against tumors, bacteria, and fungal infections (Sambasivarao, 2013; Ha et al., 2016; Balachandran and Yuana, 2019). However, there is little work published on antiviral factors that can be delivered in EVs, and most approaches involve delivery of RNA-targeting agents such as miRNAs or siRNAs. Zhang et al. (2017) report a modified method of calcium chloride-mediated transfection to introduce miRNAs in EVs, which can then be delivered efficiently *in vitro* and *in vivo*. Their method is simple, easy, and convenient. However, further work is required particularly because Zhang et al. (2017) worked on macrophages, and their efficiency of EV uptake might be high since it occurs through phagocytosis.

Zhu et al. (2014) developed a novel strategy against PRRSV infection. The available inactive and live-attenuated vaccines fail to provide sustainable protection against heterogeneous PRRSV strains. PRRSV enters porcine alveolar macrophages (PAMs) by receptor-mediated endocytosis, using heparan sulfate as the general attachment factor, sialoadhesin (Sn or CD16) for the viral binding and internalization, and CD163 for the viral genome release. Zhu et al. (2014) used artificial miRNAs (amiRNAs) targeting the viral receptors Sn and CD163 to prevent PRRSV infection. These miRNAs were packaged in EVs following transduction of pig cells with recombinant adenoviruses carrying these miRNAs. Treatment of PAMs with the two amiRNA-containing EV groups significantly inhibited PRRSV infection of PAMs. These results suggest that EVs can be used as a small RNA vehicle that can target viral receptors to decrease viral infection.

Delivery of small RNAs in EVs could also occur after infection (Bitko et al., 2005), since siRNA treatment of mice infected with RSV, even 2–3 days post infection, ameliorated pathogenesis. Therefore, EVs can be engineered as antiviral siRNA carriers, and their potency makes them attractive tools of delivery. EVs can also be used for delivery of siRNAs to a mouse brain, achieving up to 60% knockdown of the BACE1 gene (Alvarez-Erviti et al., 2011). The ability of EVs to cross the blood–brain barrier suggests that they could be used for delivery of antiviral agents in the brain, possibly targeting of viruses that either spread to the brain such as HIV-1 or establish their latent reservoir in neuronal cells in the brain such as HSV (Thompson et al., 2011; Yao et al., 2014). Delivery of therapeutic agents to the brain is a major challenge but Alvarez-Erviti et al. (2011) demonstrated the potential of EVs due to specificity and safety.

Besides targeting specific factors (e.g., viral receptors), Jeon et al. (2019) demonstrated that EVs from KSHV-infected cells (KSHV EVs) can stimulate the expression of interferon stimulated genes (ISGs) in cells exposed to KSHV EVs. Double-stranded DNA on the surface of EVs can be an inducer of inflammation, and Jeon et al., demonstrated that mtDNA is enriched in KSHV EV isolations. Knock-down of cGAS in KSHV EV-recipient cells inhibited ISGs expression, therefore it seems that mtDNA carried in KSHV EVs is sensed by cGAS in recipient cells, leading to ISGs expression. In addition, pretreatment of uninfected human umbilical vein endothelial cells (HUVECs) with KSHV EVs inhibited a subsequent infection with KSHV or HSV-1. The enhanced ISG expression observed when pre-exposing cells to mtDNA-carrying KSHV EVs is an interesting avenue for treating infections in which innate immunity is abrogated, e.g., Ebola infection (Hartman et al., 2008).

Delorme-Axford et al. (2013) focused on the resistance of the human placental trophoblasts to infection by viruses. Cultured primary human trophoblasts (PHT) are resistant to infection by a panel of viruses, and PHT conditioned media can confer that resistance to other non-PHT cell lines. The group further investigated the human cluster of C19MC miRNAs, which are almost exclusively expressed in placenta and they are the most abundant species in EVs from PHT cells. Exogenous expression of C19MC in U2OS cells conferred resistance to infections, similarly to exposure of cells to PHT EVs. Mechanistically, the miRNAs in EVs triggered autophagy, and that induction of autophagy appears to be required for resistance to viral infections. This work defines a role for EVs in autophagy-mediated antiviral responses and shows potential for a therapeutic approach when dealing with infections during pregnancy for example.

## Antiviral Effects Associated With Assorted Factors

Antiviral effects can be triggered by factors in EVs other than nucleic acids. Khatua et al. (2009) investigate the antiviral effect of APOBEC3G (A3G) that is secreted in EVs from infected cells. They used A3G expressing CD4^+^ T cells or 293T cells and demonstrated that A3G is released in EVs and is catalytically active. Pretreatment of peripheral blood mononuclear cells (PBMCs) with EVs carrying A3G followed by HIV-1 infection caused an inhibition of HIV-1 replication. Surprisingly, this happens even though the Vif protein of HIV-1, a known A3G inhibitor is expressed. It is possible that A3G in EVs has an effect before accumulation of Vif following HIV-1 infection.

Li et al. (2013) presented evidence that IFN-α induces the transfer of antiviral molecules from non-permissive liver non-parenchymal cells (LNPCs) to hepatocytes through the release of EVs. This intercellular transfer of antiviral molecules in EVs bypassed mechanisms by which the viruses counteract anti-viral responses and resulted in restoration of the antiviral state in virus-infected cells. Based on these findings, this group proposed a physiological role for EVs *in vivo* based on three points. First, inhibition of EV production by nSMase-2 or Rab27α- knockdown weakened IFN-α induced antiviral activity *in vivo*. Second, after depletion of macrophages in mice liver, a weaker antiviral response to HBV was achieved when mice liver were repopulated with BMDMs in which nSMase-2 or Rab27a were knocked down than with wild type BMDMs. Third, EVs mediated transfer of APOBEC3G from BMDMs to hepatocytes in the mouse liver after stimulation with IFNα. The delivery of strong antiviral factors in EVs may be an attractive therapeutic strategy that could lead to the development of novel treatments for chronic HBV infection and other diseases.

Kesimer et al. (2009) detected “EV-like” vesicles in human tracheobronchial epithelial (HTBE) cell culture secretions that have antiviral properties. These vesicles carried the epithelial mucins MUC1, MUC4, and MUC16, and a-2,6-linked sialic acid that was associated with these mucin molecules. The human influenza virus is known to bind sialic acid, and functional analysis of those vesicles showed that they have a neutralizing effect on influenza virus infection. Mixing EVs from HTBE cells with different doses of influenza virus resulted in dramatic loss of infectivity of the influenza virions. In addition, Kesimer et al. (2009) treated those EVs with neuraminidase, which cleaved sialic acid off the surface of EVs. Mixing the neuraminidase-treated EVs with influenza virus caused no loss of viral infectivity. These data suggest that the loss of infectivity is dependent on the interaction between the sialic acid and the virions. This work further underscores the antiviral potential of EVs, but additional work on individual pathogens is necessary to identify relevant exosomal targets.

Extracellular vesicles could also be used for the delivery of antiviral drugs. Curcumin is a phenolic compound from the spice turmeric that has a wide range of activities that include incompletely characterized antiviral activities. It can block transcription and replication of HIV-1 (Kumari et al., 2015), it promotes HIV-1 Tat degradation (Ali and Banerjea, 2016), it can abrogate HSV-2 infection if cells are pretreated with curcumin (Kutluay et al., 2008), and can lead to diminished HTLV-1 or HPV cellular transformation by inhibiting AP-1 transcription activation (Karbalaei and Keikha, 2019). It can also act as an antiherpetic compound by inhibiting p300/CBP transcription activation which is hijacked by HSV (Kutluay et al., 2008). Sun et al. (2010) demonstrate that curcumin can be incorporated in EVs, and EV-bound curcumin becomes more soluble and stable in the blood circulation of mice. Furthermore, binding of curcumin to EVs enhances its uptake by activated monocytes *in vivo*, and mice can be protected from LPS-induced septic shock. This work is a proof of principle regarding the use of EVs as a drug delivery system to combat diseases and alleviate pathogenesis. Incorporating curcumin in EVs for example, could enhance its potency *in vivo*, and could provide access to organs such as the brain that might otherwise be inaccessible due to the blood–brain barrier. Alvarez-Erviti et al. (2011) has shown that EVs can deliver cargo in the brain, and recently Morad et al. (2019) also demonstrated mechanistically how this happens. Particularly, Morad et al. (2019) demonstrated that breast cancer-derived EVs could cross the blood–brain barrier through transcytosis. The uptaken EVs are sorted in Rab11^+^ recycling endosomes and are released in the basolateral membrane. Endothelial Rab7 is downregulated, thereby preventing sorting of the uptaken EVs to lysosomes. The identification of the mechanism that EVs use to breach the blood–brain barrier can guide the development of brain-targeting therapeutics. Particularly in the case of HSV-1, the clinical use of curcumin has been investigated as a treatment for HSV-1-associated Alzheimer’s disease but curcumin’s low penetration efficiency across the blood–brain barrier is a problem (Piacentini et al., 2014). However, based on Sun et al. (2010), EVs may enable delivery of curcumin to the brain as Alvarez-Erviti et al. (2011) and Morad et al. (2019) showed.

## Vesicle-Cloaked Viruses or Viral Factors With Anti-Tumor Effects

**FIGURE 5:**
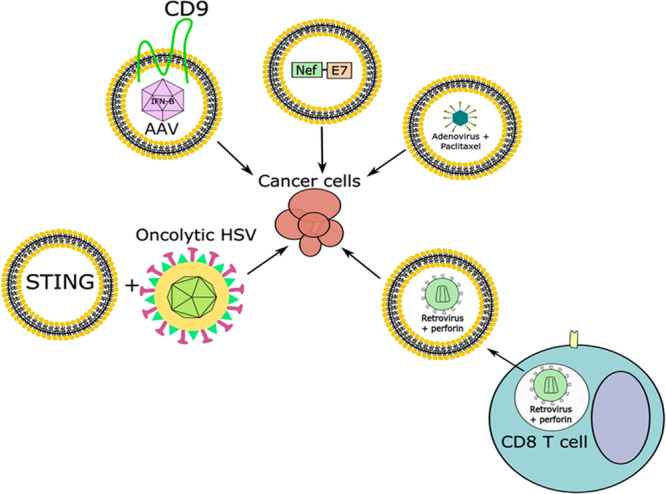
Combining EVs carrying viruses or viral factors along with viral infection to combat cancer. Adenoviruses, some retroviruses, and AAV have been found in endosomes or EVs and can be used, alone or in combination with other factors, to target cancer cells. Viral proteins can also be packaged in EVs and used for the treatment of cancer, while oncolytic viruses could be combined with EVs containing anti-tumor factors to target cancer cells.

With the recent success of different oncolytic viruses, such as recombinant measles virus, recombinant vaccinia virus, a specific serotype of reovirus, and an attenuated mutant of HSV-1 in clinical trials, there is a growing interest in modifying viruses to treat human diseases, particularly cancer. Cancer is the second leading cause of death in the United States and it is projected that the cost for cancer treatment in 2020 will be $173 billion (Mariotto et al., 2011). Therefore, there is a need for cancer treatment options. Some hurdles obstructing the use of oncolytic viruses include the targeting of these viruses to tumors, the efficiency of uptake, and the activity in the cancer cells. While in some cases oncolytic viruses may be injected directly into a tumor, this is not always the case and injecting oncolytic viruses into the bloodstream has demonstrated issues in targeting and uptake of these viruses by the tumor (Willmon et al., 2009). Therefore, one major focus for oncolytic virus research has been in improving uptake of these viruses. An approach that is currently explored is the use of EVs to deliver viruses. In one such attempt, Garofalo et al., 2018a found that when using EV-encapsulated oncolytic adenovirus there was enhanced inhibition of tumor growth *in vivo* than when using the oncolytic adenovirus alone. This inhibition was enhanced further when using EV-encapsulated paclitaxel (a drug that inhibits mitosis through stabilization of microtubules) with the oncolytic adenovirus, however, it is not fully understood how this increased anti-tumor effect was accomplished (Figure 5) (de Brabander et al., 1981; Garofalo et al., 2018a). One possibility is improved delivery. In support of this, the same group further showed that there was enhanced delivery of EV-encapsulated oncolytic adenovirus to the tumor site depending on the route of injection, and they also confirmed increased cancer cell death *in vitro* with EV-encapsulated oncolytic adenovirus compared to oncolytic adenovirus alone (Garofalo et al., 2018b). The fact that EV-encapsulated viruses display higher infectivity than free viruses was recently described for rotaviruses and noroviruses, both in cell culture models and *in vivo* (Santiana et al., 2018).

The use of adeno-associated virus (AAV) for gene therapy purposes and for the treatment of cancer is promising. However, there have been issues in using AAV for gene delivery due to pre-existing neutralizing antibodies against the virus, low levels of gene expression in target tissues, and high accumulation in the liver where AAV particles are degraded or can have toxic side effects (Murphy et al., 2019). Recently it was found that AAV can be contained in microvesicles, and these are referred to as vexosomes (Maguire et al., 2012). The vexosomes showed a greater transduction efficiency in both cultured cells and in mice than AAV alone, and they were also resistant to human neutralizing anti-AAV antibodies (Maguire et al., 2012; György et al., 2015). One successful approach utilizing AAV vexosomes (exo-AAV) is through intravascular delivery targeting tumor stromal cells in glioblastomas (Volak et al., 2018). Recently, a group used exo-AAV carrying the interferon-beta (IFN-β) gene and injected the vexosomes intravascularly, where they saw increased survival of mice that had glioblastomas compared to those treated with exo-AAV that did not contain IFN-β (Figure 5) (Volak et al., 2018). Overexpression of the tetraspanin CD9 in cells transduced with AAV increased the production of exo-AAV, and yielded a higher transduction of AAV in recipient cells than regular exo-AAV (Schiller et al., 2018). However, additional optimization is required.

Extracellular vesicles can also be used to deliver viral components. For example, EVs from HIV-1 infected cells contain the viral protein Nef (Lenassi et al., 2010). Peretti et al. (2005) had previously mutated Nef such that is was incorporated with high efficiency into virus-like particles (VLPs), while it’s C-terminus could be fused to other proteins. Both of these findings have since been built upon to include the form of Nef with high VLP incorporation efficiency fused to peptides of interest, which were found to incorporate into exosomes as efficiently as they were incorporated into VLPs (Lattanzi and Federico, 2012). Also, this group began fusing the E7 peptide from human papilloma virus (HPV) to mutated Nef and incorporating them into exosomes (Figure 5) (Di Bonito et al., 2017). HPV is an oncogenic virus, and E7 is a viral protein implicated in oncogenesis, thus developing a robust immune response against E7 could possibly prevent the development of HPV-associated cancer (Ann and Karl, 2013). This group transfected cells with a plasmid expressing the mutant form of Nef fused to HPV E7, collected and purified EVs, and inoculated mice subcutaneously. They then collected the CD8^+^ T cells from these mice and activated them with HPV E7 *in vitro*, where they saw a CD8^+^ T cell mediated cytotoxic response was elicited, which killed HPV-associated cancer cells that were pre-treated with HPV E7-positive exosomes *in vitro* (Di Bonito et al., 2015). When these exosomes were injected intramuscularly into mice with HPV-positive tumors the tumor size was smaller and weighed less than mice treated with control exosomes or exosomes containing the mutant form of Nef alone (Di Bonito et al., 2017). Therefore, this mutated form of Nef could be used to develop strategies to block tumor progression. However, there is more work needed to characterize the amount of viral protein or amount of EVs needed to accomplish such an effect without safety concerns, as well as characterization of the best immunogenic peptides to fuse to Nef. However, this strategy offers a way to utilize viral components without the need for virus, and with the flexibility of developing a more targeted approach.

Another concept is how vectors and EVs may work in tandem to induce an anti-tumor response. One example is the finding that retroviral particles loaded into T cells could be delivered to tumor cells through the endosome/lytic granule pathway (Figure 5) (Kottke et al., 2006). Endosomes are vesicles in the cytoplasm that form through endocytic trafficking pathways, and in T cells they typically contain cytotoxic/lytic granules. In this case, T cells delivered perforin and cytotoxic granules in addition to unenveloped retroviral particles to tumor cells, which increased the survival of tumor-bearing mice, including those with metastatic disease, and also protected the mice against re-challenge (Kottke et al., 2006). Another example of EVs working in tandem with a virus to modulate the intracellular environment is the observation made by Kalamvoki et al. (2014) that EVs released by HSV-1 infected cells contain STING (Figure 5). In support of a role for STING in the anti-tumor response, one group found that STING itself was down-regulated in multiple human colorectal and ovarian cancer cell lines, in addition to defects in cGAS (a cellular nucleotidyltransferase involved in the STING signaling pathway) and other stages of the STING pathway (Xia et al., 2016; De Queiroz et al., 2019). Additionally, this group found that infection of colorectal or ovarian cancer cells with the approved oncolytic HSV did not stimulate the production of IFN-β in cell lines that were shown to be defective for STING, but did cause death in multiple cell lines (Xia et al., 2016; De Queiroz et al., 2019). When mice carrying colorectal or ovarian cancer tumors with defects in the STING pathway were infected intratumorally with oncolytic HSV there were reductions in tumor size (Xia et al., 2016; De Queiroz et al., 2019). STING is of interest as a potential tumor suppressor. Characterization of EVs from an oncolytic HSV infection has not been done, but it is possible that EVs from cells infected with oncolytic HSV may contain factors which could induce an anti-tumor response by stimulating the immune system.

## Role of Evs in the Development of Viral Vaccines

Extracellular vesicles can influence both adaptive and innate immunity through the exchange of vesicles between immune cells. The idea arose when EVs released by B-cell lines were found to carry peptide/MHC class II complexes and to prime specific immune responses (Raposo et al., 1996). Exosomes from dendritic cells were also shown to carry antigen presenting molecules and accessory factors such as MHC classes I and II, CD54, and CD86 (Zitvogel et al., 1998). These EVs could induce an immune response, as antigen-specific T cell activation can be induced by EVs secreted by dendritic cells (DCs). Additionally, EVs can modulate both the viral replication and host immune response to infection by different pathogens through functioning as host and viral antigen cargo carriers (Masciopinto et al., 2004; Xu et al., 2009; Kouwaki et al., 2016, 2017; Dogrammatzis et al., 2019). EVs are an effective means of transporting cargo to recipient cells, making them ideal tools for delivering antigens. Therefore, their antigenic potential makes them ideal candidates in vaccine production.

In this area, one group (Anticoli et al., 2018) built an exosome-based platform for the generation of vaccines, aiming at the stimulation of an effective antigen-specific cytotoxic T lymphocyte (CTL) immune reaction. This platform is based on the fact that the HIV-1 Nef protein is highly incorporated into EVs, and in order to abrogate its immunomodulatory properties they have used a mutated form (Nef^mut^) that is lacking several anti-cellular effects typically induced by wild-type Nef, including CD4 down-regulation, increase of HIV-1 infectivity, and MHC class I down-regulation. This Nef^mut^ maintains its ability to incorporate into exosomes. Anticoli et al. (2018) built DNA vectors where a viral antigen of interest was fused to Nef^mut^, and subsequently this DNA vector was injected in mice intramuscularly. Viral antigens that were tested, included the Ebola virus virion proteins VP24, VP40, and nucleoprotein (NP), the influenza virus NP, the Crimean-Congo hemorrhagic fever virus NP, the West Nile virus non-structural protein 3 (NS3), and the hepatitis C virus NS3. EVs that carried Nef^mut^ fused to each viral antigen were detected in the serum of the injected mice. Also, a highly detectable CD8^+^ T cell reaction was observed for all viral proteins tested, which was specific. DNA vectors can be very stable and easy to develop, making this concept exciting. Another group (Kanuma et al., 2017), built a DNA vaccine encoding an ovalbumin (OVA) antigen fused to CD63, a tetraspanin protein that localizes to various cellular membranes including EVs. Transfection of this plasmid to cells produced OVA-carrying EVs, and immunizations with these OVA-carrying EVs primed naïve mice to induce OVA-specific CD4^+^ and CD8^+^ T cells. Therefore, the strategy of fusing antigens of interest with a protein targeted to EVs is a strategy that can mediate effective vaccine design.

PRRSV is a virus that affects pigs, with a significant economic burden. The inactivated PRRSV vaccine is safe but poorly immunogenic and has failed to prevent PRRS outbreaks and infections (Martínez-Lobo et al., 2013; Pileri et al., 2015). Strategies using nanoparticle-trapped antigens are more effective than conventional vaccine platforms (Dwivedi et al., 2013; Renukaradhya et al., 2014). To this end, one group (Montaner-Tarbes et al., 2016) determined the antigenicity of exosomes isolated from viremic or non-viremic pigs. PRRSV viral proteins were detected in exosomes using nanoscale liquid chromatography coupled to tandem mass spectrometry (nanoLC-MS/MS), particularly the RNA-dependent RNA polymerase and the highly immunogenic nucleocapsid protein N. It was further shown that exosomes that were isolated from non-viremic (NV) animals (previously infected but now free-of-virus) are antigenic when tested with sera from pigs previously exposed to PRRSV, thus establishing that viral proteins contained in the exosomes from NV animals are antigenic. This is the first report of antigenic exosomes in animals that have no detectable pathogen load and may be of importance for future vaccine approaches.

Another potential use of EVs in vaccines is as adjuvants. Qazi et al. (2009) show that OVA loaded exosomes can act as an adjuvant. They exposed DCs to OVA, and subsequently isolated exosomes that were defined as indirectly loaded, or “OVA-Exo.” They observed that co-administration *in vivo* of OVA-exo with antigen enhanced the humoral response, augmented the specific T-cell responses, and promoted a Th1-type shift in the immune response. A Th1-type shift cannot be achieved with alum or lipopolysaccharides (LPS), other commonly used adjuvants, which underlines the potential of EVs as adjuvants (Awate et al., 2013). Similarly, another group (Jesus et al., 2018) described the role of unmodified exosomes as adjuvants for hepatitis B vaccination strategies. They isolated EVs from the supernatant of LPS-stimulated human monocytic cell lines (THP-1) and showed that they evoked a pro-inflammatory profile in spleen cells of healthy mice through the induction of cytokines. Subcutaneous vaccination of mice with those EVs, combined with a solution of hepatitis B recombinant antigen (HBsAg) or a suspension containing HBsAg loaded poly-ε-caprolacone (PCL)/chitosan nanoparticles (NPs), induced a humoral immune response similar to the one induced when using HBsAg alone. However, EVs also triggered an immunomodulator effect on the cellular immune response, highlighted by the enhance of IFN-γ secretion. Interestingly, Jesus et al., detected a shift toward a Th1 response, which was observed before when using EVs as an adjuvant *in vivo* (Aline et al., 2004; Qazi et al., 2009; Jesus et al., 2018). One issue that was not addressed is whether potential LPS contamination of their isolated exosomes could be partly responsible for the immune responses. Its effect should be clarified when LPS-adjuvanted HBsAg, and LPS-free EVs are used as control samples.

## Evs as a Negative Factor in Vaccine Production

The efficacy of several vaccines can be optimized when considering the effects of EVs. The production of the rabies vaccine in Medical Research Council cell strain 5 (MRC-5) cells could be improved in terms of yield (Wang et al., 2019). MRC-5 cells have a poor susceptibility to the rabies virus (RABV) infection limiting the yield of the vaccine. EVs participate in the resistance of MRC-5 cells to RABV, through delivery of miR-423-5p. The delivery of miR-423-5p abrogates the inhibitory effect of suppressor of cytokine signaling 3 (SOCS3) on type I interferon (IFN) signaling. Targeting EV production can therefore be a potential strategy of improving MRC-5 cell-based rabies vaccine production. Additionally, miRNAs in EVs affect the response to the influenza whole-virus vaccine (WV) (Okamoto et al., 2018). miR-451a is abundant in human serum EVs and its presence in blood-circulating EVs can attenuate the innate immune response of macrophages and dendritic cells to the inactivated influenza WV. This is because EVs carrying miR-451a (and potentially other miRNAs) are internalized by macrophages and dendritic cells and can affect the immune response to WV *in vivo*. Such effects should be considered to improve current vaccine efficacy and when designing novel vaccine strategies.

Taking into consideration EVs when optimizing vaccine production is also important when working with virus-like particles (VLPs). VLPs are composed of one or more recombinantly produced structural viral proteins, which upon expression self-assemble into particles that mimic the virion structure (Roldão et al., 2010). VLPs are uptaken by antigen presenting cells (APCs) and give rise to a potent humoral and cellular immune response (Ludwig and Wagner, 2007). However, EVs and VLPs present similar physicochemical characteristics making it difficult to separate them during VLP production in any system, and often their mixing is ignored or biologically not understood (Steppert et al., 2016). As a result, VLP-based vaccine preparations contain immunologically irrelevant proteins that might affect vaccine potency. One group (Venereo-Sánchez et al., 2019) demonstrated contamination of VLPs with several host proteins due to co-purified EVs and vice versa. Based on these data, novel affinity-based methods for depletion of EVs should be developed to optimize the purity of isolated VLPs.

## Conclusion

Extracellular vesicles are natural mediators of intercellular communication, and as such they can carry RNA, proteins, and other biologically active molecules. Multiple viruses hijack EV biogenesis mechanisms to mediate their own assembly and dissemination, indicating novel antiviral targets. Such targets could be exploited to develop more effective strategies to combat viral infections or even prevent them. However, many aspects of the interplay between viruses and EV biogenesis remain to be elucidated. Before taking advantage of this relationship in a therapeutic context, we need to gain a better understanding at a foundational level to elucidate the molecular interactions. Often in the case of pathogens, characterization of EVs occurs at a single timepoint, which is a snapshot of the actual ongoing infection. One group (van der Grein et al., 2018) underlined the importance of relating EV analysis back to the relevant timepoint of the pathogen life cycle. Characterization of EVs throughout the life cycle of a pathogen of interest could provide a more precise understanding regarding the effect of the pathogen on EVs production. Spatial analysis of EVs may also be important, especially regarding therapeutics. EVs may be “edited” (van der Grein et al., 2018) depending on their milieu. Neuron-derived EVs bind Alzheimer disease-associated amyloid β-peptide, enhancing the formation of non-toxic amyloid fibrils and their uptake by microglia (Yuyama et al., 2012). EVs and retroviruses can bind to fibronectin in plasma, enhancing their uptake to target cells (Osawa et al., 2017). These and other similar EV edits should be taken into consideration when investigating the EV and pathogen interplay, or the design of EV therapeutic delivery.

Nonetheless, EVs can regulate gene expression and cellular functions in recipient cells and therefore they can cause signaling effects that can be exploited therapeutically. Therapeutic molecules, which can be otherwise difficult to deliver, can be loaded on EVs, and can then be delivered with great efficiency and efficacy. Thus, it is possible to use EVs to deliver therapeutic agents in tissues that are difficult to reach, such as past the blood–brain barrier. Also, EVs can promote the stability of compounds like curcumin, or RNA molecules with therapeutic potential like miRNAs or siRNAs. There are data showing the potential for treating viral infections using EVs, or promising work in the field of cancer therapy using EVs to trigger T cell cytotoxic responses. EVs could also be used by themselves, or in tandem with oncolytic viruses.

Although many different techniques have been utilized for the isolation of EVs in different fields, the lack of a standardized protocol to separate vesicles from virions in viral infections makes clinical uses of such protocols complicated (Gyorgy et al., 2015; Murphy et al., 2019). Additionally, EVs can have diverse routes of uptake and a better understanding is necessary to improve delivery of functional cargo in the tissue of interest (Mathieu et al., 2019; Murphy et al., 2019). Strategies like engineering EVs to carry ligands on their surface that can bind to receptors in a particular tissue, which provides a targeted approach, have shown potential (Lattanzi and Federico, 2012; Di Bonito et al., 2017). However, more work is necessary to correlate EVs uptake with an effect. Many experiments have been performed using very different number of EVs. Generally, EV yield per cell varies depending on the cell line and its physiological status. Most studies suggest an average yield of 1,000–2,000 EVs per cell. It is unclear how physiologically relevant are experiments that describe EV effects by exposing cells at a lot higher numbers of EVs or based on the amount of protein. Additionally, such experiments describe variable durations of EV exposure or multiple waves. Therefore, further research is required to determine appropriate conditions.

Promising work has been done in the field of vaccine development. EVs can carry viral proteins and can be immunogenic when administered *in vivo*. However, the immunological effect of altered EV cargo during an infection can be both pro-viral and anti-viral. Therefore, further research is required in order to discern the cargo with antiviral properties that could be exploited for delivery. EVs can also impose a risk and complication for vaccine production. Their shared physicochemical properties with most viruses might result in contamination of vaccine preparations, thereby withholding the full immunogenic potential of vaccine antigens. More sensitive separation techniques of EVs could prevent such problems.

Besides the similar physicochemical properties, it is intriguing that viruses and EVs utilize common machinery for their assembly and release. Similarly to how HIV Gag co-opts the ESCRT machinery for HIV release, two groups (Ashley et al., 2018; Pastuzyn et al., 2018) show how the neuronal Gag-like Arc protein can form virus-like capsids that can bind its own mRNA and traffic across synapses. Similar behavior was observed with the Gag region of the Copia retrotransposon (a distantly Arc-related retroelement) that could form a complex with its own mRNA. The plethora of such retroelements in eukaryotic genomes suggests that they may utilize EV biogenesis mechanisms for intercellular transfer. Since such retroelements are considered ancestral to modern retroviruses (Malik et al., 2000), it is an intriguing question whether those elements co-opted EV biogenesis to become infectious and gave genesis to retroviruses, or whether they domesticated already existing viral mechanisms to shuttle across cells.

Overall, the interwoven relationships between viruses and EVs have indicated novel areas of research with therapeutic potential.

## Author Contributions

MK is the principal investigator and provided support and guidance. CD and HW have contributed equally to this work.

## Conflict of Interest

The authors declare that the research was conducted in the absence of any commercial or financial relationships that could be construed as a potential conflict of interest.

## References

[B1] Ali A. Banerjea A. C. (2016). Curcumin inhibits HIV-1 by promoting tat protein degradation. *Sci. Rep.* 6 1–9. 10.1038/srep27539 27283735 PMC4901322

[B2] Aline F. Bout D. Amigorena S. Roingeard P. Dimier-Poisson I. (2004). *Toxoplasma gondii* antigen-pulsed-dendritic cell-derived exosomes induce a protective immune response against *T. gondii* infection. *Infect. Immun.* 72 4127–4137. 10.1128/IAI.72.7.4127-4137.2004 15213158 PMC427397

[B3] Alvarez-Erviti L. Seow Y. Yin H. Betts C. Lakhal S. Wood M. J. A. (2011). Delivery of SiRNA to the mouse brain by systemic injection of targeted exosomes. *Nat. Biotechnol.* 29 306–309. 10.1038/nbt.1807 21423189

[B4] Ann R. Karl M. (2013). The papillomavirus E7 proteins. *Virology* 445 138–168. 10.1016/j.virol.2013.04.013 23731972 PMC3783579

[B5] Anticoli S. Manfredi F. Chiozzini C. Arenaccio C. Olivetta E. Ferrantelli F. (2018). An exosome-based vaccine platform imparts cytotoxic T lymphocyte immunity against viral antigens. *Biotechnol. J.* 13 1–7. 10.1002/biot.201700443 29274250

[B6] Arii J. Watanabe M. Maeda F. Tokai-Nishizumi N. Chihara T. Miura M. (2018). ESCRT-III mediates budding across the inner nuclear membrane and regulates its integrity. *Nat. Commun.* 9:3379. 10.1038/s41467-018-05889-9 30139939 PMC6107581

[B7] Ashley J. Cordy B. Lucia D. Fradkin L. G. Budnik V. Thomson T. (2018). Retrovirus-like gag protein Arc1 binds RNA and traffics across synaptic boutons. *Cell* 172 262.e11–274.e11. 10.1016/j.cell.2017.12.022 29328915 PMC5793882

[B8] Awate S. Babiuk L. A. Mutwiri G. (2013). Mechanisms of action of adjuvants. *Front. Immunol.* 4:114. 10.3389/fimmu.2013.00114 23720661 PMC3655441

[B9] Balachandran B. Yuana Y. (2019). Extracellular vesicles-based drug delivery system for cancer treatment. *Cogent Med.* 6 1–23. 10.1080/2331205x.2019.1635806

[B10] Barger J. F. Rahman M. A. Jackson D. Acunzo M. Nana-Sinkam S. P. (2016). Extracellular MiRNAs as biomarkers in cancer. *Food Chem. Toxicol.* 98 66–72. 10.1016/j.fct.2016.06.010 27311798 PMC5086286

[B11] Bello-Morales R. Praena B. de la Nuez C. Rejas M. T. Guerra M. Galán-Ganga M. (2018). Role of microvesicles in the spread of herpes simplex virus 1 in oligodendrocytic cells. *J. Virol.* 92 1–19. 10.1128/jvi.00088-18 29514899 PMC5923088

[B12] Bharat T. A. M. Noda T. Riches J. D. Kraehling V. Kolesnikova L. Becker S. (2012). Structural dissection of ebola virus and its assembly determinants using cryo-electron tomography. *Proc. Natl. Acad. Sci. U.S.A.* 109 4275–4280. 10.1073/pnas.1120453109 22371572 PMC3306676

[B13] Bhatnagar S. Schorey J. S. (2007). Exosomes released from infected macrophages contain mycobacterium avium glycopeptidolipids and are proinflammatory. *J. Biol. Chem.* 282 25779–25789. 10.1074/jbc.M702277200 17591775 PMC3636815

[B14] Bissig C. Gruenberg J. (2014). ALIX and the multivesicular endosome: ALIX in wonderland. *Trends Cell Biol.* 24 19–25. 10.1016/j.tcb.2013.10.009 24287454

[B15] Bitko V. Musiyenko A. Shulyayeva O. Barik S. (2005). Inhibition of respiratory viruses by nasally administered SiRNA. *Nat. Med.* 11 50–55. 10.1038/nm1164 15619632

[B16] Bukong T. N. Hou W. Kodys K. Szabo G. (2013). Ethanol facilitates HCV replication via upregulation of GW182 and HSP90 in human hepatoma cells. *Hepatology* 57 70–80. 22898980 10.1002/hep.26010 PMC3540130

[B17] Bukong T. N. Momen-Heravi F. Kodys K. Bala S. Szabo G. (2014). Exosomes from hepatitis C infected patients transmit HCV infection and contain replication competent viral RNA in complex with Ago2-MiR122-HSP90. *PLoS Pathog.* 10:e1004424. 10.1371/journal.ppat.1004424 25275643 PMC4183590

[B18] Chahar H. S. Corsello T. Kudlicki A. S. Komaravelli N. Casola A. (2018). Respiratory syncytial virus infection changes cargo composition of exosome released from airway epithelial cells. *Sci. Rep.* 8 1–18. 10.1038/s41598-017-18672-5 29321591 PMC5762922

[B19] de Brabander M. Geuens G. Nuydens R. Willebrords R. De Mey J. (1981). Taxol induces the assembly of free microtubules in living cells and blocks the organizing capcity of the centrosomes and kinetochores. *Proc. Natl. Acad. Sci. U.S.A.* 78 5608–5612. 10.1073/pnas.78.9.5608 6117858 PMC348802

[B20] De Queiroz N. M. G. P. Xia T. Konno H. Barber G. N. (2019). Ovarian cancer cells commonly exhibit defective STING signaling which affects sensitivity to viral oncolysis. *Mol. Cancer Res.* 17 974–986. 10.1158/1541-7786.MCR-18-0504 30587523 PMC6445711

[B21] Deatheragea B. L. Cooksona B. T. (2012). Membrane vesicle release in bacteria, eukaryotes, and archaea: a conserved yet underappreciated aspect of microbial life. *Infect. Immun.* 80 1948–1957. 10.1128/IAI.06014-11 22409932 PMC3370574

[B22] Delorme-Axford E. Donker R. B. Mouillet J. F. Chu T. Bayer A. Ouyang Y. (2013). Human placental trophoblasts confer viral resistance to recipient cells. *Proc. Natl. Acad. Sci. U.S.A.* 110 12048–12053. 10.1073/pnas.1304718110 23818581 PMC3718097

[B23] Deng L. Jiang W. Wang X. Merz A. Hiet M. S. Chen Y. (2019). Syntenin regulates hepatitis C virus sensitivity to neutralizing antibody by promoting E2 secretion through exosomes. *J. Hepatol.* 71 52–61. 10.1016/j.jhep.2019.03.006 30880226

[B24] Deschamps T. Kalamvoki M. (2018). Extracellular vesicles released by herpes simplex virus 1 infected cells block virus replication in recipient cells in a STING-dependent manner. *J. Virol.* 92:e01102-18. 10.1128/JVI.01102-18 29976662 PMC6146713

[B25] Di Bonito P. Chiozzini C. Arenaccio C. Anticoli S. Manfredi F. Olivetta E. (2017). Antitumor HPV E7-specific CTL activity elicited by *in vivo* engineered exosomes produced through DNA inoculation. *Int. J. Nanomed.* 12 4579–4591. 10.2147/IJN.S131309 28694699 PMC5491702

[B26] Di Bonito P. Ridolfi B. Columba-Cabezas S. Giovannelli A. Chiozzini C. Manfredi F. (2015). HPV-E7 delivered by engineered exosomes elicits a protective CD8+ T Cell-mediated immune response. *Viruses* 7 1079–1099. 10.3390/v7031079 25760140 PMC4379561

[B27] Dogrammatzis C. Deschamps T. Kalamvoki M. (2019). Biogenesis of extracellular vesicles during herpes simplex 1 infection: role of the CD63 tetraspanin. *J. Virol.* 93 1–15. 10.1128/JVI.01850-18 30355691 PMC6321934

[B28] Du Y. Xu Y. Ding L. Yao H. Yu H. Zhou T. (2009). Down-regulation of MiR-141 in gastric cancer and its involvement in cell growth. *J. Gastroenterol.* 44 556–561. 10.1007/s00535-009-0037-7 19363643

[B29] Dwivedi V. Manickam C. Binjawadagi B. Renukaradhya G. J. (2013). PLGA nanoparticle entrapped killed porcine reproductive and respiratory syndrome virus vaccine helps in viral clearance in pigs. *Vet. Microbiol.* 166 47–58. 10.1016/J.VETMIC.2013.04.029 23764272 PMC7117126

[B30] Elgner F. Ren H. Medvedev R. Ploen D. Himmelsbach K. Boller K. (2016). The intracellular cholesterol transport inhibitor U18666A inhibits the exosome-dependent release of mature hepatitis C virus. *J. Virol.* 90 11181–11196. 10.1128/jvi.01053-16 27707921 PMC5126375

[B31] Emerson S. U. Nguyen H. T. Torian U. Burke D. Engle R. Purcell R. H. (2010). Release of genotype 1 hepatitis E virus from cultured hepatoma and polarized intestinal cells depends on open reading frame 3 protein and requires an intact PXXP motif. *J. Virol.* 84 9059–9069. 10.1128/jvi.00593-10 20610720 PMC2937629

[B32] Feng Z. Hensley L. McKnight K. L. Hu F. Madden V. Ping L. (2013). A pathogenic picornavirus acquires an envelope by hijacking cellular membranes. *Nature* 496 367–371. 10.1038/nature12029 23542590 PMC3631468

[B33] Flanagan J. Middeldorp J. Sculley T. (2003). Localization of the epstein-barr virus protein LMP 1 to exosomes. *J. Gen. Virol.* 84 1871–1879. 10.1099/vir.0.18944-0 12810882

[B34] Garofalo M. Saari H. Somersalo P. Crescenti D. Kuryk L. Aksela L. (2018a). Antitumor effect of oncolytic virus and paclitaxel encapsulated in extracellular vesicles for lung cancer treatment. *J. Control. Release* 283 223–234. 10.1016/j.jconrel.2018.05.015 29864473

[B35] Garofalo M. Villa A. Rizzi N. Kuryk L. Mazzaferro V. Ciana P. (2018b). Systemic administration and targeted delivery of immunogenic oncolytic adenovirus encapsulated in extracellular vesicles for cancer therapies. *Viruses* 10:558. 10.3390/v10100558 30322158 PMC6213631

[B36] González-López O. Rivera-Serrano E. E. Hu F. Hensley L. McKnight K. L. Ren J. (2018). Redundant late domain functions of tandem VP2 YPX 3 L motifs in nonlytic cellular egress of quasi-enveloped hepatitis a virus. *J. Virol.* 92 1–16. 10.1128/jvi.01308-18 PMC6232465 30232181

[B37] Gould S. J. Booth A. M. Hildreth J. E. K. (2003). The trojan exosome hypothesis. *Proc. Natl. Acad. Sci. U.S.A.* 100 10592–10597. 10.1073/pnas.1831413100 12947040 PMC196848

[B38] György B. Fitzpatrick Z. Crommentuijn M. H. W. Mu D. (2015). Naturally enveloped AAC vectors for sheilding neutralising antibodies and robust gene delivery *in vivo*. *Biomaterials* 35 7598–7609. 10.1016/j.biomaterials.2014.05.032.Naturally PMC4104587 24917028

[B39] Gyorgy B. Hung M. E. Breakefield X. O. Leonard J. N. (2015). Therapeutic application of extracellular vesicles: clinical promise and open questions. *Annu. Rev. Pharmacol. Toxicol.* 55 439–464. 10.1016/j.physbeh.2017.03.040 25292428 PMC4445965

[B40] Ha D. Yang N. Nadithe V. (2016). Exosomes as therapeutic drug carriers and delivery vehicles across biological membranes: current perspectives and future challenges. *Acta Pharm. Sin. B* 6 287–296. 10.1016/j.apsb.2016.02.001 27471669 PMC4951582

[B41] Han Z. Liu X. Chen X. Zhou X. Du T. Roizman B. (2016). MiR-H28 and MiR-H29 expressed late in productive infection are exported and restrict HSV-1 replication and spread in recipient cells. *Proc. Natl. Acad. Sci. U.S.A.* 113 E894–E901. 10.1073/pnas.1525674113 26831114 PMC4763765

[B42] Hartman A. L. Ling L. Nichol S. T. Hibberd M. L. (2008). Whole-genome expression profiling reveals that inhibition of host innate immune response pathways by ebola virus can be reversed by a single amino acid change in the VP35 protein. *J. Virol.* 82 5348–5358. 10.1128/jvi.00215-08 18353943 PMC2395193

[B43] Henke J. I. Goergen D. Zheng J. Song Y. Schüttler C. G. Fehr C. (2008). MicroRNA-122 stimulates translation of hepatitis C virus RNA. *EMBO J.* 27 3300–3310. 10.1038/emboj.2008.244 19020517 PMC2586803

[B44] Honegger A. Leitz J. Bulkescher J. Hoppe-Seyler K. Hoppe-Seyler F. (2013). Silencing of human papillomavirus (HPV) E6/E7 oncogene expression affects both the contents and the amounts of extracellular microvesicles released from HPV-positive cancer cells. *Int. J. Cancer* 133 1631–1642. 10.1002/ijc.28164 23526637

[B45] Honegger A. Schilling D. Bastian S. Sponagel J. Kuryshev V. Sültmann H. (2015). Dependence of intracellular and exosomal MicroRNAs on Viral E6/E7 oncogene expression in HPV-positive tumor cells. *PLoS Pathog.* 11:e1004712. 10.1371/journal.ppat.1004712 25760330 PMC4356518

[B46] Huang R. Wu J. Zhou X. Jiang H. Zhou G. G. Roizman B. (2019). HSV-1 MiR-H28 exported to uninfected cells in exosomes restricts cell-to-cell virus spread by inducing IFNγ MRNAs. *J. Virol.* 93:e01005-19 10.1128/jvi.01005-19 PMC6803268 31413129

[B47] Hurwitz S. N. Nkosi D. Conlon M. M. York S. B. Liu X. Tremblay D. C. (2017). CD63 regulates epstein-barr virus LMP1 exosomal packaging, enhancement of vesicle production, and noncanonical NF-K B signaling. *J. Virol.* 91:e02251-16 10.1128/JVI.02251-16 PMC5309960 27974566

[B48] Jaiswal R. Sedger L. M. (2019). Intercellular vesicular transfer by exosomes, microparticles and oncosomes - implications for cancer biology and treatments. *Front. Oncol.* 9:125 10.3389/fonc.2019.00125 PMC6414436 30895170

[B49] Jeon H. Lee J. Lee S. Kang S. K. Park S. J. Yoo S. M. (2019). Extracellular vesicles from KSHV-infected cells stimulate antiviral immune response through mitochondrial DNA. *Front. Immunol.* 10:876 10.3389/fimmu.2019.00876 PMC6491682 31068945

[B50] Jesus S. Soares E. Cruz M. T. Borges O. (2018). Exosomes as adjuvants for the recombinant hepatitis B antigen: first report. *Eur. J. Pharm. Biopharm.* 133 1–11. 10.1016/J.EJPB.2018.09.029 30287267

[B51] Jia X. Chen J. Megger D. A. Zhang X. Kozlowski M. Zhang L. (2017). Label-free proteomic analysis of exosomes derived from inducible hepatitis B virus-replicating HepAD38 cell line. *Mol. Cell. Proteom.* 16 S144–S160. 10.1074/mcp.M116.063503 PMC5393393 28242843

[B52] Joshi A. Munshi U. Ablan S. D. Nagashima K. Freed E. O. (2008). Functional replacement of a retroviral late domain by ubiquitin fusion. *Traffic* 9 1972–1983. 10.1111/j.1600-0854.2008.00817.x 18817521 PMC2763539

[B53] Kakizaki M. Yamamoto Y. Yabuta S. Kurosaki N. Kagawa T. Kotani A. (2018). The immunological function of extracellular vesicles in hepatitis B virus-infected hepatocytes. *PLoS One* 13:e0205886 10.1371/journal.pone.0205886 PMC6312312 30596665

[B54] Kalamvoki M. Deschamps T. (2016). Extracellular vesicles during herpes simplex virus type 1 infection: an inquire. *Virol. J.* 13 1–12. 10.1186/s12985-016-0518-2 27048572 PMC4822280

[B55] Kalamvoki M. Du T. Roizman B. (2014). Cells infected with herpes simplex virus 1 export to uninfected cells exosomes containing STING, viral MRNAs, and MicroRNAs. *Proc. Natl. Acad. Sci. U.S.A.* 111 E4991–E4996. 10.1073/pnas.1419338111 25368198 PMC4246290

[B56] Kanuma T. Yamamoto T. Kobiyama K. Moriishi E. Masuta Y. Kusakabe T. (2017). CD63-mediated antigen delivery into extracellular vesicles via DNA vaccination results in robust CD8 + T cell responses. *J. Immunol.* 198 4707–4715. 10.4049/jimmunol.1600731 28507029

[B57] Kapoor N. R. Chadha R. Kumar S. Choedon T. Reddy V. S. Kumar V. (2017). The HBx gene of hepatitis B virus can influence hepatic microenvironment via exosomes by transferring its MRNA and protein. *Virus Res.* 240 166–174. 10.1016/j.virusres.2017.08.009 28847700

[B58] Karbalaei M. Keikha M. (2019). Curcumin as an Herbal Inhibitor Candidate Against HTLV-1 protease. *Jentashapir J. Health Res.* 10 1–4. 10.5812/jjhr.92813

[B59] Kesimer M. Scull M. Brighton B. DeMaria G. Burns K. O’Neal W. (2009). Characterization of exosome-like vesicles released from human tracheobronchial ciliated epithelium: a possible role in innate defense. *FASEB J.* 23 1858–1868. 10.1096/fj.08-119131 19190083 PMC2698655

[B60] Khan S. Aspe J. R. Asumen M. G. Almaguel F. Odumosu O. Acevedo-Martinez S. (2009). Extracellular, cell-permeable survivin inhibits apoptosis while promoting proliferative and metastatic potential. *Br. J. Cancer* 100 1073–1086. 10.1038/sj.bjc.6604978 19293795 PMC2669990

[B61] Khan S. Jutzy J. M. Aspe J. R. McGregor D. W. Neidigh J. W. Wall N. R. (2011). Survivin is released from cancer cells via exosomes. *Apoptosis* 16 1–12. 10.1007/s10495-010-0534-4 20717727 PMC3174681

[B62] Khatua A. K. Taylor H. E. Hildreth J. E. K. Popik W. (2009). Exosomes packaging APOBEC3G confer human immunodeficiency virus resistance to recipient cells. *J. Virol.* 83 512–521. 10.1128/jvi.01658-08 18987139 PMC2612372

[B63] Klesney-Tait J. I Turnbull R. Colonna M. (2006). The TREM receptor family and signal integration. *Nat. Immunol.* 7 1266–1273. 10.1038/ni1411 17110943

[B64] Komabayashi Y. Kishibe K. Nagato T. Ueda S. Takahara M. Harabuchi Y. (2017). Circulating epstein-barr virus–encoded micro-RNAs as potential biomarkers for nasal natural Killer/T-cell lymphoma. *Hematol. Oncol.* 35 655–663. 10.1002/hon.2360 27709652

[B65] Kosaka N. Iguchi H. Yoshioka Y. Takeshita F. Matsuki Y. Ochiya T. (2010). Secretory mechanisms and intercellular transfer of MicroRNAs in living cells. *J. Biol. Chem.* 285 17442–17452. 10.1074/jbc.M110.107821 20353945 PMC2878508

[B66] Kottke T. Qiao J. Diaz R. M. Ahmed A. Vroman B. Thompson J. (2006). The perforin-dependent immunological synapse allows T-cell activation-dependent tumor targeting by MLV vector particles. *Gene Ther.* 13 1166–1177. 10.1038/sj.gt.3302722 16625245

[B67] Kouwaki T. Fukushima Y. Daito T. Sanada T. Yamamoto N. Mifsud E. J. (2016). Extracellular vesicles including exosomes regulate innate immune responses to hepatitis B virus infection. *Front. Immunol.* 7:335 10.3389/fimmu.2016.00335 PMC5005343 27630638

[B68] Kouwaki T. Okamoto M. Tsukamoto H. Fukushima Y. Oshiumi H. (2017). Extracellular Vesicles Deliver Host and Virus RNA and regulate innate immune response. *Int. J. Mol. Sci.* 18 1–11. 10.3390/ijms18030666 PMC5372678 28335522

[B69] Kowal J. Tkach M. Théry C. (2014). Biogenesis and secretion of exosomes. *Curr. Opin. Cell Biol.* 29 116–125. 10.1016/j.ceb.2014.05.004 24959705

[B70] Kumari N. Kulkarni A. A. Lin X. McLean C. Ammosova T. Ivanov A. (2015). Inhibition of HIV-1 by curcumin A, a novel curcumin analog. *Drug Des. Dev. Ther.* 9 5051–5060. 10.2147/DDDT.S86558 PMC4562762 26366056

[B71] Kutluay S. B. Doroghazi J. Roemer M. E. Triezenberg S. J. (2008). Curcumin-inhibits herpes simplex virus immediate-early gene expression by a mechanism independent of P300/CBP histone acetyltransferase activity. *Virology* 373 239–247. 10.1038/jid.2014.371 18191976 PMC2668156

[B72] Lang F. M. Hossain A. Gumin J. Momin E. N. Shimizu Y. Ledbetter D. (2018). Mesenchymal stem cells as natural biofactories for exosomes carrying Mir-124a in the treatment of gliomas. *Neuro Oncol.* 20 380–390. 10.1093/neuonc/nox152 29016843 PMC5817945

[B73] Lattanzi L. Federico M. (2012). A strategy of antigen incorporation into exosomes: comparing cross-presentation levels of antigens delivered by engineered exosomes and by lentiviral virus-like particles. *Vaccine* 30 7229–7237. 10.1016/j.vaccine.2012.10.010 23099330

[B74] Lee J. H. Schierer S. Blume K. Dindorf J. Wittki S. Xiang W. (2016). HIV-Nef and ADAM17-containing plasma extracellular vesicles induce and correlate with immune pathogenesis in chronic HIV infection. *EBioMedicine* 6 103–113. 10.1016/j.ebiom.2016.03.004 27211553 PMC4856776

[B75] Lenassi M. Cagney G. Liao M. Vaupotič T. Bartholomeeusen K. Cheng Y. (2010). HIV Nef is secreted in exosomes and triggers apoptosis in bystander CD4+ T cells. *Traffic* 11 110–122. 10.1111/j.1600-0854.2009.01006.x 19912576 PMC2796297

[B76] Li J. Liu K. Liu Y. Xu Y. Zhang F. Yang H. (2013). Exosomes mediate the cell-to-cell transmission of IFN-α-induced antiviral activity. *Nat. Immunol.* 14 793–803. 10.1038/ni.2647 23832071

[B77] Liang B. Song Y. Zheng W. Ma W. (2016). MiRNA143 induces K562 cell apoptosis through downregulating BCR-ABL. *Med. Sci. Monit.* 22 2761–2767. 10.12659/MSM.895833 27492780 PMC4978212

[B78] Lin S. L. Chiang A. Chang D. Ying S. Y. (2008). Loss of Mir-146a function in hormone-refractory prostate cancer. *RNA* 14 417–424. 10.1261/rna.874808 18174313 PMC2248249

[B79] Lorizate M. Kräusslich H. G. (2011). Role of lipids in virus replication. *Cold Spring Harb. Perspect. Biol.* 3 1–20. 10.1101/cshperspect.a004820 PMC3179339 21628428

[B80] Lorizate M. Sachsenheimer T. Glass B. Habermann A. Gerl M. J. Kräusslich H. G. (2013). Comparative lipidomics analysis of HIV-1 particles and their producer cell membrane in different cell lines. *Cell. Microbiol.* 15 292–304. 10.1111/cmi.12101 23279151

[B81] Ludwig C. Wagner R. (2007). Virus-like particles-universal molecular toolboxes. *Curr. Opin. Biotechnol.* 18 537–545. 10.1016/j.copbio.2007.10.013 18083549 PMC7126091

[B82] Madison M. N. Okeoma C. M. (2015). Exosomes: implications in HIV-1 pathogenesis. *Viruses* 7 4093–4118. 10.3390/v7072810 26205405 PMC4517139

[B83] Maguire C. A. Balaj L. Sivaraman S. Crommentuijn M. H. W. Ericsson M. Mincheva-Nilsson L. (2012). Microvesicle-associated AAV vector as a novel gene delivery system. *Mol. Ther.* 20 960–971. 10.1038/mt.2011.303 22314290 PMC3345986

[B84] Malik H. S. Henikoff S. Eickbush T. H. (2000). Poised for contagion: evolutionary origins of the infectious abilities of invertebrate retroviruses. *Genome Res.* 10 1307–1318. 10.1101/gr.145000 10984449

[B85] Mantzorou M. Pavlidou E. Vasios G. Tsagalioti E. Giaginis C. (2018). Effects of curcumin consumption on human chronic diseases: a narrative review of the most recent clinical data. *Phytother. Res.* 32 957–975. 10.1002/ptr.6037 29468820

[B86] Mao L. Wu J. Shen L. Yang J. Chen J. Xu H. (2016). Enterovirus 71 transmission by exosomes establishes a productive infection in human neuroblastoma cells. *Virus Genes* 52 189–194. 10.1007/s11262-016-1292-3 26837894

[B87] Mariotto A. B. Robin Yabroff K. Shao Y. Feuer E. J. Brown M. L. (2011). Projections of the cost of cancer care in the United States: 2010-2020. *J. Natl. Cancer Instit.* 103 117–128. 10.1093/jnci/djq495 PMC3107566 21228314

[B88] Martínez-Lobo F. J. De Lome L. C. íez-Fuertes F. D. Segalés J. García-Artiga C. Simarro I. (2013). Safety of porcine reproductive and respiratory syndrome modified live virus (MLV) vaccine strains in a young pig infection model. *Vet. Res.* 44 1–14. 10.1186/1297-9716-44-115 24308693 PMC4028782

[B89] Masciopinto F. Giovani C. Campagnoli S. Galli-Stampino L. Colombatto P. Brunetto M. (2004). Association of hepatitis C virus envelope proteins with exosomes. *Eur. J. Immunol.* 34 2834–2842. 10.1002/eji.200424887 15368299

[B90] Mathieu M. Martin-Jaular L. Lavieu G. Théry C. (2019). Specificities of secretion and uptake of exosomes and other extracellular vesicles for cell-to-cell communication. *Nat. Cell Biol.* 21 9–17. 10.1038/s41556-018-0250-9 30602770

[B91] McNamara R. P. Costantini L. M. Myers T. A. Schouest B. Maness N. J. Griffith J. D. (2018). Nef secretion into extracellular vesicles or exosomes is conserved across human and simian immunodeficiency viruses. *mBio* 9 1–20. 10.1128/mBio.02344-17 PMC5801467 29437924

[B92] Mills J. T. Schwenzer A. Marsh E. K. Edwards M. R. Sabroe I. Midwood K. S. (2019). Airway epithelial cells generate pro-inflammatory tenascin-C and small extracellular vesicles in response to TLR3 stimuli and rhinovirus infection. *Front. Immunol.* 10:1987 10.3389/fimmu.2019.01987 PMC6712508 31497021

[B93] Mitchell P. S. Parkin R. K. Kroh E. M. Fritz B. R. Wyman S. K. Pogosova-Agadjanyan E. L. (2008). Circulating MicroRNAs as stable blood-based markers for cancer detection. *Proc. Natl. Acad. Sci. U.S.A.* 105 10513–10518. 10.1073/pnas.0804549105 18663219 PMC2492472

[B94] Montaner-Tarbes S. Borrás F. E. Montoya M. Fraile L. Del Portillo H. A. (2016). Serum-derived exosomes from non-viremic animals previously exposed to the porcine respiratory and reproductive virus contain antigenic viral proteins. *Vet. Res.* 47 1–10. 10.1186/s13567-016-0345-x 26738942 PMC4702310

[B95] Morad G. Carman C. V. Hagedorn E. J. Perlin J. R. Zon L. I. Mustafaoglu N. (2019). Tumor-derived extracellular vesicles breach the intact blood–brain barrier via transcytosis. *ACS Nano* 13 13853–13865. 10.1021/acsnano.9b04397 31479239 PMC7169949

[B96] Mori Y. Koike M. Moriishi E. Kawabata A. Tang H. Oyaizu H. (2008). Human herpesvirus-6 induces MVB formation, and virus egress occurs by an exosomal release pathway. *Traffic* 9 1728–1742. 10.1111/j.1600-0854.2008.00796.x 18637904 PMC2613231

[B97] Mukhamedova N. Hoang A. Dragoljevic D. Dubrovsky L. Pushkarsky T. Low H. (2019). Exosomes containing HIV protein nef reorganize lipid rafts potentiating inflammatory response in bystander cells. *PLoS Pathog.* 15:e1007907 10.1371/journal.ppat.1007907 PMC6657916 31344124

[B98] Murphy D. E. de Jong O. G. Brouwer M. Wood M. J. Lavieu G. Schiffelers R. M. (2019). Extracellular vesicle-based therapeutics: natural versus engineered targeting and trafficking. *Exp. Mol. Med.* 51 1–12. 10.1038/s12276-019-0223-5 PMC6418170 30872574

[B99] Nagashima S. Takahashi M. Kobayashi T. Nishizawa T. Nishiyama T. Primadharsini P. P. (2017). Characterization of the quasi-enveloped hepatitis E virus particles released by the cellular exosomal pathway. *J. Virol.* 91 1–16. 10.1128/jvi.00822-17 PMC5660490 28878075

[B100] Noda T. Hagiwara K. Sagara H. Kawaoka Y. (2010). Characterization of the ebola virus nucleoprotein-RNA complex. *J. Gen. Virol.* 91 1478–1483. 10.1099/vir.0.019794-0 20164259 PMC2878588

[B101] Okamoto M. Fukushima Y. Kouwaki T. Daito T. Kohara M. Kida H. (2018). MicroRNA-451a in extracellular, blood-resident vesicles attenuates macrophage and dendritic cell responses to influenza whole-virus vaccine. *J. Biol. Chem.* 293 18585–18600. 10.1074/jbc.RA118.003862 30282637 PMC6290151

[B102] Olmos Y. Carlton J. (2016). The ESCRT machinery: new roles at new holes. *Curr. Opin. Cell Biol.* 38 1–11. 10.1016/J.CEB.2015.12.001 26775243 PMC5023845

[B103] Osawa S. Kurachi M. Yamamoto H. Yoshimoto Y. Ishizaki Y. (2017). Fibronectin on extracellular vesicles from microvascular endothelial cells is involved in the vesicle uptake into oligodendrocyte precursor cells. *Biochem. Biophys. Res. Commun.* 488 232–238. 10.1016/j.bbrc.2017.05.049 28499870

[B104] Pap E. Pállinger É Pásztói M. Falus A. (2009). Highlights of a new type of intercellular communication: microvesicle-based information transfer. *Inflamm. Res.* 58 1–8. 10.1007/s00011-008-8210-7 19132498

[B105] Pastuzyn E. D. Day C. E. Kearns R. B. Kyrke-Smith M. Taibi A. V. McCormick J. (2018). The neuronal gene arc encodes a repurposed retrotransposon gag protein that mediates intercellular RNA transfer. *Cell* 172 275.e18–288.e18. 10.1016/j.cell.2017.12.024 29328916 PMC5884693

[B106] Pawliczek T. Crump C. M. (2009). Herpes simplex virus Type 1 production requires a functional ESCRT-III complex but is independent of TSG101 and ALIX expression. *J. Virol.* 83 11254–11264. 10.1128/jvi.00574-09 19692479 PMC2772754

[B107] Pegtel D. M. Cosmopoulos K. Thorley-Lawson D. A. Van Eijndhoven M. A. J. Hopmans E. S. Lindenberg J. L. (2010). Functional delivery of viral MiRNAs via exosomes. *Proc. Natl. Acad. Sci. U.S.A.* 107 6328–6333. 10.1073/pnas.0914843107 20304794 PMC2851954

[B108] Peretti S. Schiavoni I. Pugliese K. Federico M. (2005). Cell death induced by the herpes simplex virus-1 thymidine kinase delivered by human immunodeficiency virus-1-based virus-like particles. *Mol. Ther.* 12 1185–1196. 10.1016/j.ymthe.2005.06.474 16095973

[B109] Piacentini R. De Chiara G. Domenica D. L. P. Ripoli C. Marcocci M. E. Garaci E. (2014). HSV-1 and Alzheimer’s disease: more than a hypothesis. *Front. Pharmacol.* 5:97 10.3389/fphar.2014.00097 PMC4019841 24847267

[B110] Piccin A. Murphy W. G. Smith O. P. (2007). Circulating microparticles: pathophysiology and clinical implications. *Blood Rev.* 21 157–171. 10.1016/j.blre.2006.09.001 17118501

[B111] Pileri E. Gibert E. Soldevila F. García-Saenz A. Pujols J. Diaz I. (2015). Vaccination with a genotype 1 modified live vaccine against porcine reproductive and respiratory syndrome virus significantly reduces viremia, viral shedding and transmission of the virus in a quasi-natural experimental model. *Vet. Microbiol.* 175 7–16. 10.1016/j.vetmic.2014.11.007 25439650

[B112] Pleet M. L. DeMarino C. Stonier S. W. Dye J. M. Jacobson S. Aman M. J. (2019). Extracellular vesicles and ebola virus: a new mechanism of immune evasion. *Viruses* 11:410 10.3390/v11050410 PMC6563240 31052499

[B113] Pleet M. L. Erickson J. Demarino C. Barclay R. A. Cowen M. Lepene B. (2018). Ebola virus VP40 modulates cell cycle and biogenesis of extracellular vesicles. *J. Infect. Dis.* 218 S365–S387. 10.1093/infdis/jiy472 30169850 PMC6249571

[B114] Pleet M. L. Mathiesen A. DeMarino C. Akpamagbo Y. A. Barclay R. A. Schwab A. (2016). Ebola VP40 in exosomes can cause immune cell dysfunction. *Front. Microbiol.* 7:1765 10.3389/fmicb.2016.01765 PMC5098130 27872619

[B115] Qazi K. R. Gehrmann U. Jordö E. D. Karlsson M. C. I. Gabrielsson S. (2009). Antigen-loaded exosomes alone induce Thl-type memory through a B cell dependent mechanism. *Blood* 113 2673–2683. 10.1182/blood-2008-04-153536 19176319

[B116] Ramakrishnaiah V. Thumann C. Fofana I. Habersetzer F. Pan Q. De Ruiter P. E. (2013). Exosome-mediated transmission of hepatitis C virus between human hepatoma Huh7.5 cells. *Proc. Natl. Acad. Sci. U.S.A.* 110 13109–13113. 10.1073/pnas.1221899110 23878230 PMC3740869

[B117] Raposo G. Nijman H. W. Stoorvogel W. Leijendekker R. Hading C. V. Melief C. J. M. (1996). B lymphocytes secrete antigen-presenting vesicles. *J. Exp. Med.* 183 1161–1172. 10.1084/jem.183.3.1161 8642258 PMC2192324

[B118] Raposo G. Stoorvogel W. (2013). Extracellular vesicles: exosomes, microvesicles, and friends. *J. Cell Biol.* 200 373–383. 10.1083/jcb.201211138 23420871 PMC3575529

[B119] Raymond A. D. Campbell-Sims T. C. Khan M. Lang M. Huang M. B. Bond V. C. (2011). HIV type 1 Nef is released from infected cells in CD45+ microvesicles and is present in the plasma of HIV-infected individuals. *AIDS Res. Hum. Retroviruses* 27 167–178. 10.1089/aid.2009.0170 20964480 PMC3064529

[B120] Renukaradhya G. Binjawadagi B. Dwivedi V. Manickam C. Ouyang K. Torrelles J. (2014). An innovative approach to induce cross-protective immunity against porcine reproductive and respiratory syndrome virus in the lungs of pigs through adjuvanted nanotechnology-based vaccination. *Int. J. Nanomed.* 9:1519 10.2147/IJN.S59924 PMC3969340 24711701

[B121] Robinson S. M. Tsueng G. Sin J. Mangale V. Rahawi S. Mcintyre L. L. (2014). Coxsackievirus B exits the host cell in shed microvesicles displaying autophagosomal markers. *PLoS Pathog.* 10:e1004045 10.1371/journal.ppat.1004045 PMC3983045 24722773

[B122] Roldão A. Leda R. Jt M. (2010). Virus-like particles in vaccine development. *Expert Rev. Vacc.* 9 1149–1176. 10.1586/erv.10.115 20923267

[B123] Ronghua L. Xiaoyu F. Yujing T. Lei F. Deming T. Yi O. (2018). Expression profiles of the exosomal mirnas in the chronic hepatitis B patients with persistently normal ALT. *J. Central S. Univ.* 43 475–480. 10.11817/j.issn.1672-7347.2018.05.003 29886461

[B124] Sadeghipour S. Mathias R. A. (2017). Herpesviruses hijack host exosomes for viral pathogenesis. *Semin. Cell Dev. Biol.* 67 91–100. 10.1016/j.semcdb.2017.03.005 28456604

[B125] Sambasivarao S. V. (2013). NIH public access. *Curr. Rheumatol. Rep.* 18 1199–1216. 10.1016/j.micinf.2011.07.011.Innate

[B126] Santiana M. Ghosh S. Ho B. A. Rajasekaran V. Du W. L. Mutsafi Y. (2018). Vesicle-cloaked virus clusters are optimal units for inter-organismal viral transmission. *Cell Host Microbe* 24 208.e8–220.e8. 10.1016/j.chom.2018.07.006 30092198 PMC6226266

[B127] Schiller L. T. Lemus-Diaz N. Rinaldi Ferreira R. Böker K. O. Gruber J. (2018). Enhanced production of exosome-associated AAV by overexpression of the tetraspanin CD9. *Mol. Ther. Methods Clin. Dev.* 9 278–287. 10.1016/j.omtm.2018.03.008 29707602 PMC5918177

[B128] Schorey J. S. Cheng Y. Singh P. P. Smith V. L. (2015). Exosomes and other extracellular vesicles in host–pathogen interactions. *EMBO Rep.* 16 24–43. 10.15252/embr.201439363 25488940 PMC4304727

[B129] Shinozaki A. Sakatani T. Ushiku T. Hino R. Isogai M. Ishikawa S. (2010). Downregulation of MicroRNA-200 in EBV-associated gastric carcinoma. *Cancer Res.* 70 4719–4727. 10.1158/0008-5472.CAN-09-4620 20484038

[B130] Shtam T. Naryzhny S. Kopylov A. Petrenko E. Samsonov R. Kamyshinsky R. (2018). Functional properties of circulating exosomes mediated by surface-attached plasma proteins. *J. Hematol.* 7 149–153. 10.14740/jh412w 32300430 PMC7155850

[B131] Skalsky R. L. Cullen B. R. (2010). Viruses, MicroRNAs, and host interactions. *Annu. Rev. Microbiol.* 64 123–141. 10.1146/annurev.micro.112408.134243 20477536 PMC3621958

[B132] Skog J. Würdinger T. van Rijn S. Meijer D. H. Gainche L. Curry W. T. (2008). Glioblastoma microvesicles transport RNA and proteins that promote tumour growth and provide diagnostic biomarkers. *Nat. Cell Biol.* 10 1470–1476. 10.1038/ncb1800 19011622 PMC3423894

[B133] Steppert P. Burgstaller D. Klausberger M. Berger E. Aguilar P. P. Schneider T. A. (2016). Purification of HIV-1 Gag virus-like particles and separation of other extracellular particles. *J. Chromatogr. A* 1455 93–101. 10.1016/j.chroma.2016.05.053 27286649

[B134] Stoorvogel W. Kleijmeer M. J. Geuze H. J. Raposo G. (2002). The biogenesis and functions of exosomes. *Traffic* 3 321–330. 10.1034/j.1600-0854.2002.30502.x 11967126

[B135] Strack B. Calistri A. Gottlinger H. G. (2002). Late assembly domain function can exhibit context dependence and involves ubiquitin residues implicated in endocytosis. *J. Virol.* 76 5472–5479. 10.1128/jvi.76.11.5472-5479.2002 11991975 PMC137019

[B136] Sun D. Zhuang X. Xiang X. Liu Y. Zhang S. Liu C. (2010). A novel nanoparticle drug delivery system: the anti-inflammatory activity of curcumin is enhanced when encapsulated in exosomes. *Mol. Ther.* 18 1606–1614. 10.1038/mt.2010.105 20571541 PMC2956928

[B137] Tai Y. L. Chen K. C. Hsieh J. T. Shen T. L. (2018). Exosomes in cancer development and clinical applications. *Cancer Sci.* 109 2364–2374. 10.1111/cas.13697 29908100 PMC6113508

[B138] Tamai K. Shiina M. Tanaka N. Nakano T. Yamamoto A. Kondo Y. (2012). Regulation of hepatitis C virus secretion by the Hrs-dependent exosomal pathway. *Virology* 422 377–385. 10.1016/j.virol.2011.11.009 22138215

[B139] Tan R. Z. Liu J. Zhang Y. Y. Wang H. L. Li J. C. Liu Y. H. (2019). Curcumin relieved cisplatin-induced kidney inflammation through inhibiting mincle-maintained M1 macrophage phenotype. *Phytomedicine* 52 284–294. 10.1016/j.phymed.2018.09.210 30599909

[B140] Taylor D. D. Gercel-Taylor C. (2008). MicroRNA signatures of tumor-derived exosomes as diagnostic biomarkers of ovarian cancer. *Gynecol. Oncol.* 110 13–21. 10.1016/j.ygyno.2008.04.033 18589210

[B141] Taylor M. P. Burgon T. B. Kirkegaard K. Jackson W. T. (2009). Role of microtubules in extracellular release of poliovirus. *J. Virol.* 83 6599–6609. 10.1128/jvi.01819-08 19369338 PMC2698579

[B142] Temme S. Eis-Hübinger A. M. McLellan A. D. Koch N. (2010). The herpes simplex virus-1 encoded glycoprotein B diverts HLA-DR into the exosome pathway. *J. Immunol.* 184 236–243. 10.4049/jimmunol.0902192 19949097

[B143] Théry C. Amigorena S. Raposo G. Clayton A. (2006). Isolation and characterization of exosomes from cell culture supernatants and biological fluids. *Curr. Protoc. Cell Biol.* 30 3.22.1–3.22.29. 10.1002/0471143030.cb0322s30 18228490

[B144] Théry C. Zitvogel L. Amigorena S. (2002). Exosomes: composition, biogenesis and function. *Nat. Rev. Immunol.* 2 569–579. 10.1038/nri855 12154376

[B145] Thompson K. A. Cherry C. L. Bell J. E. McLean C. A. (2011). Brain cell reservoirs of latent virus in presymptomatic HIV-infected individuals. *Am. J. Pathol.* 179 1623–1629. 10.1016/j.ajpath.2011.06.039 21871429 PMC3181362

[B146] Tricarico C. Clancy J. D’Souza-Schorey C. (2017). Biology and biogenesis of shed microvesicles. *Small GTPases* 8 220–232. 10.1080/21541248.2016.1215283 27494381 PMC5680703

[B147] Ushijima Y. Koshizuka T. Goshima F. Kimura H. Nishiyama Y. (2008). Herpes simplex virus type 2 UL56 interacts with the ubiquitin ligase Nedd4 and increases its ubiquitination. *J. Virol.* 82 5220–5233. 10.1128/jvi.02515-07 18353951 PMC2395212

[B148] Valadi H. Ekström K. Bossios A. Sjöstrand M. Lee J. J. Lötvall J. O. (2007). Exosome-mediated transfer of MRNAs and MicroRNAs is a novel mechanism of genetic exchange between cells. *Nat. Cell Biol.* 9 654–659. 10.1038/ncb1596 17486113

[B149] van der Grein S. G. Defourny K. A. Y. Slot E. F. J. Nolte-’t Hoen E. N. M. (2018). Intricate relationships between naked viruses and extracellular vesicles in the crosstalk between pathogen and host. *Semin. Immunopathol.* 40 491–504. 10.1007/s00281-018-0678-9 29789863 PMC6208671

[B150] van Dongen H. M. Masoumi N. Witwer K. W. Pegtel D. M. (2016). Extracellular vesicles exploit viral entry routes for cargo delivery. *Microbiol. Mol. Biol. Rev.* 80 369–386. 10.1128/mmbr.00063-15 26935137 PMC4867369

[B151] van Niel G. Charrin S. Simoes S. Romao M. Rochin L. Saftig P. (2011). The tetraspanin CD63 regulates ESCRT-independent and -dependent endosomal sorting during melanogenesis. *Dev. Cell* 21 708–721. 10.1016/j.devcel.2011.08.019 21962903 PMC3199340

[B152] Van Niel G. D’Angelo G. Raposo G. (2018). Shedding light on the cell biology of extracellular vesicles. *Nat. Rev. Mol. Cell Biol.* 19 213–228. 10.1038/nrm.2017.125 29339798

[B153] Venereo-Sánchez A. Fulton K. Koczka K. Twine S. Chahal P. Ansorge S. (2019). Characterization of influenza H1N1 Gag virus-like particles and extracellular vesicles co-produced in HEK-293SF. *Vaccine* 37 7100–7107. 10.1016/j.vaccine.2019.07.057 31358407

[B154] Volak A. LeRoy S. G. Natasan J. S. Park D. J. Cheah P. S. Maus A. (2018). Virus vector-mediated genetic modification of brain tumor stromal cells after intravenous delivery. *J. Neuro Oncol.* 139 293–305. 10.1007/s11060-018-2889-2 PMC6454875 29767307

[B155] Votteler J. Sundquist W. I. (2013). Virus budding and the ESCRT pathway. *Cell Host Microbe* 14 232–241. 10.1016/j.chom.2013.08.012 24034610 PMC3819203

[B156] Walker J. D. Maier C. L. Pober J. S. (2009). Cytomegalovirus-infected human endothelial cells can stimulate allogeneic CD4 + Memory T cells by releasing antigenic exosomes. *J. Immunol.* 182 1548–1559. 10.4049/jimmunol.182.3.1548 19155503 PMC2630120

[B157] Wang J. Teng Y. Zhao G. Li F. Hou A. Sun B. (2019). Exosome-mediated delivery of inducible Mir-423-5p enhances resistance of Mrc-5 cells to rabies virus infection. *Int. J. Mol. Sci.* 20:1537 10.3390/ijms20071537 PMC6479321 30934732

[B158] Wang L. Chen X. Zhou X. Roizman B. Zhou G. G. (2018). MiRNAs targeting ICP4 and delivered to susceptible cells in exosomes block HSV-1 replication in a dose-dependent manner. *Mol. Ther.* 26 1032–1039. 10.1016/j.ymthe.2018.02.016 29526650 PMC6080130

[B159] Willmon C. Harrington K. Kottke T. Prestwich R. Melcher A. Vile R. (2009). Cell carriers for oncolytic viruses: fed Ex for cancer therapy. *Mol. Ther.* 17 1667–1676. 10.1038/mt.2009.194 19690519 PMC2834999

[B160] Wilson J. A. Zhang C. Huys A. Richardson C. D. (2011). Human Ago2 is required for efficient MicroRNA 122 regulation of hepatitis C virus RNA accumulation and translation. *J. Virol.* 85 2342–2350. 10.1128/jvi.02046-10 21177824 PMC3067765

[B161] Wurdinger T. Gatson N. N. Balaj L. Kaur B. Breakefield X. O. Pegtel D. M. (2012). Extracellular vesicles and their convergence with viral pathways. *Adv. Virol.* 2012:767694 10.1155/2012/767694 PMC3410301 22888349

[B162] Xia T. Konmo H. Ahn J. Barber G. N. (2016). Deregulation of STING signaling in colorectal carcinoma constrains DNA-damage responses and correlates with tumorigenesis. *Cell Rep.* 14 282–297. 26748708 10.1016/j.celrep.2015.12.029 PMC4845097

[B163] Xu W. Santini P. A. Sullivan J. S. He B. Shan M. Ball S. C. (2009). HIV-1 evades virus-specific IgG2 and IgA responses by targeting systemic and intestinal B cells via long-range intercellular conduits. *Nat. Immunol.* 10 1008–1017. 10.1038/ni.1753 19648924 PMC2784687

[B164] Yao H.-W. Ling P. Tung Y.-Y. Hsu S.-M. Chen S.-H. (2014). *In vivo* reactivation of latent herpes simplex virus 1 in mice can occur in the brain before occurring in the trigeminal ganglion. *J. Virol.* 88 11264–11270. 10.1128/jvi.01616-14 25031345 PMC4178801

[B165] Yuyama K. Sun H. Mitsutake S. Igarashi Y. (2012). Sphingolipid-modulated exosome secretion promotes clearance of amyloid-β by microglia. *J. Biol. Chem.* 287 10977–10989. 10.1074/jbc.M111.324616 22303002 PMC3322859

[B166] Zeelenberg I. S. Ostrowski M. Krumeich S. Bobrie A. Jancic C. Boissonnas A. (2008). Targeting tumor antigens to secreted membrane vesicles in vivo induces efficient antitumor immune responses. *Cancer Res.* 68 1228–1235. 10.1158/0008-5472.CAN-07-3163 18281500

[B167] Zhang D. Lee H. Zhu Z. Minhas J. K. Jin Y. (2016). Enrichment of selective MiRNAs in exosomes and delivery of exosomal MiRNAs *in vitro* and *in vivo*. *Am. J. Physiol. Lung Cell. Mol. Physiol.* 312 L110–L121. 10.1152/ajplung.00423.2016 27881406 PMC5283929

[B168] Zhang H.-L. Ye H.-Q. Liu S.-Q. Deng C.-L. Li X.-D. Shi P.-Y. (2017). West nile virus NS1 antagonizes interferon-β production by targeting RIG-I and MDA5. *J. Virol.* 91:e02396-16 10.1128/JVI.02396-16 PMC5571242 28659477

[B169] Zhang Z. Yu X. Zhou Z. Li B. Peng J. Wu X. (2019). LMP1-positive extracellular vesicles promote radioresistance in nasopharyngeal carcinoma cells through P38 MAPK signaling. *Cancer Med.* 8 6082–6094. 10.1002/cam4.2506 31436393 PMC6792483

[B170] Zhou W. Woodson M. Neupane B. Bai F. Sherman M. B. Choi K. H. (2018). Exosomes serve as novel modes of tick-borne flavivirus transmission from arthropod to human cells and facilitates dissemination of viral RNA and proteins to the vertebrate neuronal cells. *PLoS Pathog.* 14:e1006764 10.1371/journal.ppat.1006764 PMC5754134 29300779

[B171] Zhu L. Song H. Zhang X. Xia X. Sun H. (2014). Inhibition of porcine reproductive and respiratory syndrome virus infection by recombinant adenovirus- and/or exosome-delivered the artificial MicroRNAs targeting sialoadhesin and CD163 receptors. *Virol. J.* 11:225 10.1186/s12985-014-0225-9 PMC4279792 25522782

[B172] Zicari S. Arakelyan A. Palomino R. A. Ñ Fitzgerald W. Vanpouille C. Lebedeva A. (2018). Human cytomegalovirus-infected cells release extracellular vesicles that carry viral surface proteins. *Virology* 524 97–105. 10.1016/j.virol.2018.08.008 30165311 PMC6258833

[B173] Zitvogel L. Regnault A. Lozier A. Wolfers J. Flament C. Tenza D. (1998). Eradication of established murine tumors usinga novel cell-free vaccine: dendritic cell-derived exosomes. *Nat. Med.* 4 594–600. 10.1038/nm0598-594 9585234

[B174] Zou X. Yuan M. Zhang T. Wei H. Xu S. Jiang N. (2019). Extracellular vesicles expressing a single-chain variable fragment of an HIV-1 specific antibody selectively target Env + tissues. *Theranostics* 9 5657–5671. 10.7150/thno.33925 31534509 PMC6735399

